# Trust or money? Barriers to health and healthcare behavior during the COVID-19 pandemic

**DOI:** 10.1371/journal.pone.0331600

**Published:** 2025-09-10

**Authors:** Caroline Rudisill, Katie Linvill, Anna Chupak, Joan Costa-Font, Sayward Harrison, Peiyin Hung, Xiaoming Li

**Affiliations:** 1 Department of Health Promotion, Education and Behavior, Arnold School of Public Health, University of South Carolina, Columbia, South Carolina, United States of America; 2 Department of Physics & Astronomy, University of Denver, Denver, Colorado, United States of America; 3 Department of Health Policy, London School of Economics & Political Science, London, United Kingdom; 4 Department of Psychology, College of Arts and Sciences, University of South Carolina, Columbia, South Carolina, United States of America; 5 Supporting Substance Use Disorder Services in South Carolina (SSUDS-SC) Center, University of South Carolina, Columbia, South Carolina, United States of America; 6 Department of Health Services Policy and Management, Arnold School of Public Health, University of South Carolina, Columbia, South Carolina, United States of America; 7 South Carolina Rural and Minority Health Research Center, University of South Carolina, Columbia, South Carolina, United States of America; 8 USC Big Data Health Science Center, University of South Carolina, Columbia, South Carolina, United States of America; 9 SC SmartState Center for Healthcare Quality, University of South Carolina, Columbia, South Carolina, United States of America; Yonsei University Medical Center: Yonsei University Health System, KOREA, REPUBLIC OF

## Abstract

This study aimed to examine how trust in institutions and changes in household finances were associated with healthcare utilization and preventive behaviors during and immediately after the COVID-19 pandemic. The COVID-19 pandemic worsened health disparities, ignited distrust in healthcare systems, and contributed to household economic shifts for many United States (US) residents. To examine these issues, we surveyed a nationally representative sample of US residents in July 2020 (n = 1,085) and May 2023 (n = 2,189). These repeated cross-sectional surveys enabled investigation of how trust in key stakeholders (e.g., federal government, the healthcare system) and household finances were linked with various types of healthcare utilization (e.g., annual preventive visits, receipt of pharmacy-based healthcare), preventive health care (e.g., influenza vaccination), and preventive behaviors (e.g., exercise, healthy eating). In 2023, the likelihoods of using some types of healthcare (annual health check and pharmacy-based healthcare) and engaging in preventive health behaviors increased relative to 2020. Improved household finances were associated with greater odds of healthy eating, exercising, and receiving annual preventive visits. Trust in the healthcare system was positively associated with all healthcare use types examined including preventive care such as influenza immunization and the individual prevention behavior of healthy eating but not exercise. Findings highlight the important role healthcare systems can have as trusted entities in potentially supporting healthcare utilization and prevention in the post-pandemic environment. Policy implications of these findings include increased efforts by payers and healthcare systems to facilitate positive health behaviors for US residents via specific strategies, such as making annual preventive health checks more accessible. At the same time, it is critical to support maintaining and building trust in healthcare systems to promote appropriate healthcare utilization.

## Introduction

The COVID-19 pandemic brought a shock both to individual lives and broader systems in the United States (US) and across the globe [1]. Societal shifts have occurred since the pandemic’s onset (e.g., increase in remote work, masking during times of high viral transmission). There were also major impacts on household finances through COVID-19-related morbidity and mortality, labor market shifts (e.g., layoffs), and stimulus payments [2–4]. Given the environment of risk and uncertainty inherent in a pandemic, trust in institutions responsible for managing pandemic response is likely to play an important role in individuals’ behaviors during and immediately post-pandemic [5–8].

The COVID-19 pandemic rapidly changed individuals’ interactions with health systems, including through shifts in public health policy, pandemic-related impacts to healthcare service availability, and socio-behavioral factors (e.g., changes in attitudes, perceptions, and behaviors). Restrictions on in-person services, including both preventive and elective care, resulted in reductions in healthcare use overall [9], even with waivers for telehealth services and increased telehealth use in general [10]. Also, uptake of pharmacy-based care increased, particularly for COVID-19 testing and vaccination, due to regulatory and reimbursement changes [11]. After the pandemic, some shifts appear to have lasted. There is enhanced capacity for telehealth [12] as well as reimbursement changes [13,14] to finance such care delivery.

There is also evidence that people’s likelihood of engaging in health-related behaviors changed during the COVID-19 pandemic. The COVID-19 pandemic is generally considered to have had negative impacts on healthy eating [15] and exercise [16]. Influenza vaccination rates also may have had spillover effects from COVID-19 vaccination rates such that during the period September 2021- January 2022, states with higher COVID-19 vaccine uptake also experienced higher flu vaccine uptake, and similarly, states with lower COVID-19 vaccine uptake saw lower flu vaccine uptake in comparison to the flu vaccination season before the COVID-19 pandemic (September 2019 to January 2020) [17].

### The role of trust in using healthcare and uptake of preventive behaviors

Trust in healthcare systems constitutes an integral element of determining how individuals use and interact with those systems, as well as participate in recommended health behaviors such as heathy eating and vaccination. Trust is key to positive health outcomes [18] and has been shown to predict health behaviors, such as childhood [19] and COVID-19 vaccinations [20–22]. Moreover, trust is associated with following advice from healthcare professionals [23,24] and accepting differing care modalities such as telehealth [25] and mobile health (mHealth) [26]. These issues have been brought to the forefront by the pandemic [8], particularly regarding whether people trust in and follow advice from healthcare professionals.

#### Changes in household finances and healthcare use and health-related behaviors.

Shifts in household finances during and immediately after the COVID-19 pandemic also likely influenced interactions with healthcare systems and health-related behaviors. Some patients with COVID-19 experienced a significant economic burden due to increased medical costs [27,28]. As emergency benefits ended, some US residents experienced a post-pandemic healthcare affordability crisis [29]. Moreover, the pandemic brought economic burdens related to job loss, inflation, and shifts in childcare responsibilities resulting in changing workforce involvement [4,30,31].

Individual and household finances also play a key role in health behaviors. Healthy eating, diet quality, and income are linked, such that those with lower income are more likely to have poorer diet quality partly due to the cost of nutrient-dense foods relative to less nutritious alternatives [32], with variation in this finding by race and ethnicity [33]. Exercise has also been shown to be associated with income [34,35] though this relationship is complex [36]. For instance, individuals with lower incomes have lower rates of sedentary behavior, and those with higher income undertake less frequent but more intense exercise and are more likely to meet daily physical activity guidelines [36]. Therefore, we expect that exercise and healthy eating may be less likely to occur in households experiencing financial difficulty during and immediately post-COVID-19.

### Conceptual framework

Health care utilization behaviors are considered to be driven both by individual and systems-level factors. For instance, Andersen and Newman’s model of determinants of healthcare utilization provides a useful framework to examine these relationships. Briefly, this model conceptualizes healthcare utilization as determined by health service delivery components (e.g., resources and their organization), medical technology and patterns of care (e.g., hospital vs outpatient setting), and individual-level determinants (predisposing – e.g., age, gender, beliefs; enabling – e.g., income, insurance status, urban/rural; illness level – e.g., diagnoses, symptoms) (37). Our focus on trust in healthcare systems aligns with predisposing factors while changes in household finances is an enabling factor [37]. Our study brings a dynamism to this theoretical model by examining likelihood of healthcare utilization during and immediately after the COVID-19 pandemic (i.e., 2020 and 2023, respectively). While this model does not specifically conceptualize preventive health behaviors examined in this study, its depiction of predisposing and enabling factors is relevant for health behaviors.

### Novel contributions of this study

To our knowledge, no existing analysis has examined the roles of changes in trust in key stakeholders and household finances, both of which were affected by the COVID-19 pandemic, in shaping a variety of healthcare utilization and preventive health behaviors. Understanding how shocks from the pandemic (e.g., loss of economic security, reduced confidence in institutions) may have impacted key health-related behaviors is critical for shaping public policy, specifically encouraging healthcare access and utilization and health promoting behaviors post-pandemic. Therefore, this study aimed to address three questions: (1) How did healthcare utilization and preventive health behaviors change over the COVID-19 pandemic for US residents? (2) How has trust in key institutional stakeholders changed during and immediately after the COVID-19 pandemic? (3) Are changes in trust in key stakeholders and household finances since the COVID-19 pandemic associated with shifts in healthcare utilization and preventive health behaviors?

To answer these questions, we conducted a repeated cross-sectional nationally representative survey of US residents in July 2020 and May 2023. Participants reported on numerous healthcare behaviors, including utilization of healthcare modalities that expanded during the pandemic (e.g., telehealth, pharmacy-based healthcare services). We controlled for household income separately from changes in household finances over the pandemic period, which allowed for isolation of the pandemic’s specific effects on household budgets regardless of income.

## Methods

### Data

We ran two surveys (repeated cross-sections) with a total sample size of 3,274 using the Ipsos Global Online Omnibus. The surveys included individuals 18−75 years of age residing in the US. The first survey (n = 1,085) was conducted from July 10−14, 2020, when COVID-19 cases were generally trending down after the initial surge [38], stay-at-home orders (e.g., lockdowns) and other restrictions were beginning to loosen, and the COVID-19 vaccine was not yet available. The second survey ran from May 24−29, 2023 (n = 2,189). This was after both the World Health Organization (WHO) [39] and the US Department of Health and Human Services [40] ended the global and federal public health emergencies (i.e., May 5, 2023 and May 11, 2023, respectively) and COVID-19 vaccines had been widely available for over two years.

Ipsos surveyed a nationally representative quota (non-probability) sample from their existing Ipsos panel with quotas set for age, gender, US region, and working status. Representativeness was also achieved on the quota dimensions using survey weights. Weights supplied by Ipsos accounted for proportions of the population who are not online and thus ineligible for this survey by gender within age and working status, household income and region. Panel members were vetted by Ipsos with quality checks to ensure that participants were unique and had the characteristics they stated in the survey. These included fraud checks, verification of location of internet protocol (IP) address and validation via CAPTCHA security code [41]. Ipsos sets sampling targets through extensive efforts that include matching with census data and a proprietary process that balances respondents asked to participate based on the target population.

Invitations to participate were sent via email or an app until sample quotas were met. Once a quota was met, individuals in that quota could no longer respond but additional surveys might be sent to specific quota groups to meet sample requirements. Non-respondents received one reminder about survey completion. Continued non-response resulted in replacement from that quota sample based on a ‘first come first complete’ basis in which the survey would close for individuals from the panel once quota samples were reached. The survey response rate was 75%.

Surveys were self-administered using respondents’ preferred method (e.g., smartphone, computer). Protocols to detect and remove respondents who completed the survey too quickly (i.e., ≥ 3 times faster than median) and straight-line responses (i.e., repeated same responses) were employed by Ipsos for quality control. Questions common to both surveys appear in S1 File, and respondents’ demographic information (e.g., gender, age) was supplied by Ipsos. Ipsos administered financial incentives using a point system for survey completion as part of their standard procedures (41). Other findings from these surveys have been published previously [42–44]. The study protocol was approved by the University of South Carolina Institutional Review Board for Human Research as exempt. Participants provided consent to be part of the overall Ipsos panel, and the IRB did not require additional consent since it was conducted through an existing global online omnibus panel.

### Survey items

Survey items were developed by the study team and/or informed by existing measures with adaptation to the COVID-19 context. As part of our process in developing the surveys, we examined existing COVID-19 surveys, some of which had validated or previously existing measures, including the PhenX Toolkit for COVID-19 [45]. We also reviewed the broader literature outside of COVID-19 studies for relevant measures or question structures. Where possible we adapted existing questions to the study context and always used standard response scales. Questions were also reviewed and commented upon by Ipsos’ survey design experts for responsiveness and clarity.

We had four items for self-reported changes in healthcare utilization (annual health check, telephone-based healthcare consultation, virtual/remote healthcare consultation such as an ‘e-visit’, telehealth virtual doctor visit or remote diagnosis and treatment, pharmacy-based healthcare such as for vaccination or testing services) and three items for preventive health behavior (healthy eating, exercise, seasonal influenza vaccination).

Participants were asked about healthcare utilization and preventive health behaviors with the prompt, ‘Since the outbreak of COVID-19, are you more or less likely to do each of the following or is there no difference?’ with Likert scale responses of ‘much more likely’, ‘somewhat more likely’, ‘no difference’, ‘somewhat less likely’, ‘much less likely’, ‘not applicable’, ‘don’t know’, and ‘prefer not to say’.

These questions are framed as ‘since the outbreak of COVID-19’ to focus on pandemic-related changes and rely on the pandemic’s salience to respondents. Available survey measures at the time of our first survey development in May-June 2020 were designed to focus on health behaviors during the COVID-19 pandemic. A question from the NIH Common Data Elements repository that came from a draft question bank of the CDC’s COVID-19 Community Survey via the All of Us Research Program [46] provided a foundation to guide our question development. This question prompt began, ‘To cope with the social distancing and isolation, are you doing any of the following?’ and included three items with yes/no responses: ‘eating more food than usual, ‘eating less food than usual’ and ‘contacting a healthcare provider’. We wanted to be precise in terms of COVID-19 impacts and not assume social distancing and isolation in our sample, so we reframed the question prompt. In addition, the standard Likert scale used in the present study provided the nuance desired for our study’s objectives. Prompts for our question included a selection of healthcare utilization types motivated by the goal to examine more novel modalities of healthcare increasingly common during the pandemic (i.e., telephone, virtual/remote and pharmacy) and key types of preventive health actions that might be affected by the pandemic (i.e., healthy eating, exercise, seasonal influenza vaccination).

Trust in stakeholders was elicited via the following question – ‘To what extent do you trust or not trust the way the following are dealing with the COVID-19 pandemic?’. This question is similar to a ‘trust in government’ question used by Gallup since the 1970s [47] but includes stakeholders relevant to this specific study. Stakeholders listed include ‘your national [federal] government officials,’ ‘your local government officials, ‘the healthcare system’ and the ‘World Health Care Organization (WHO).’ Respondents could select ‘trust a great deal, trust a fair amount, do not trust very much, do not trust at all or don’t know’, responses used by Gallup [47] except we used ‘don’t know’ rather than ‘no opinion’ to be similar to other survey questions. These stakeholders were chosen because of their critical roles in COVID-19 response. For example, local governments were often at the forefront of responses affecting people’s daily lives (e.g., business and school closures).

For household finances, at the time of our first survey development in May-June 2020, we found several questions that used percentages to ask respondents the likelihood of negative economic outcomes (e.g., running out of money because of the coronavirus) [48]. To move away from asking respondents for percentage responses, we developed a question with standard Likert responses to ask respondents to describe their household finances at the time of the survey compared to before the pandemic; ‘Which, if any, of the following would you say best describes your household finances?’ Possible responses were ‘Our household finances now are much better than before the COVID-19 pandemic,’ ‘…a little better…,’ ‘…a little worse…, ‘…much worse…,’ ‘…no difference…,’ ‘don’t know’ and ‘prefer not to say.’ Like available questions at the time, it focused on the financial effects of the COVID-19 pandemic but with a standard response scale.

We also elicited self-reported physical and mental health status based on questions from the Patient-Reported Outcomes Measurement Information System (PROMIS) but with examples specific to study goals [49]. For physical health, we asked ‘How is your physical health (e.g., pain, disease) in general?’, and for mental health, ‘How is your mental health (e.g., anxiety, depression, stress, eating disorders)?’. At wave two, participants were asked again, “How would you say [your physical health, your mental health] is now?’. Responses were ‘very good,’ ‘good,’ ‘fair,’ ‘poor,’ ‘very poor,’ ‘don’t know,’ ‘prefer not to say.’ Control variables supplied by Ipsos were gender, age, household income, education, marital status, children in household (i.e., < 18 years of age), residence rurality (i.e., urban/rural) and region).

### Statistical analysis

Our empirical strategy assumed that changes in healthcare utilization and preventive health behaviors are dependent on observed and unobserved factors. Let ψ_i_ stand for likelihood of behavior since the pandemic (annual health check, telephone-based healthcare consultation, virtual/remote healthcare consultation, pharmacy-based care, healthy eating, exercise, seasonal influenza vaccination) with specification at the individual level (_i_). Our main interest is the effect of trust in key stakeholders (ρ_i_) as a vector of trust in each stakeholder (federal government, local government, healthcare system, WHO), and household finances (λ_i_) on likelihood of behavior (ψ_i_). Models also include study year (ν_i_), a vector of self-reported health status to include physical and mental health (ρ_i_) and controls (χ_i_) (gender, age, household income, education, marital status, children in household, residence rurality, and region). The error term (ε) accounts for unobservables not specified. Our specification contains many observed individual-level determinants and some patterns of care (e.g., rural residence) as in Andersen and Newman [37]; however, many variables are left unobserved due to survey length limitations. The full estimation is as below.


$$\psi_{\text{i}}=\beta_{0}+\rho_{\text{i}}\beta_{1}+\lambda_{\text{i}}\beta_{2}+\text{\textbackslash{} }\nu_{\text{i}}\beta_{3}+\rho_{\text{i}}\beta_{4}+\chi_{\text{i}}\beta_{5}+\epsilon$$

We estimated differences in responses for dependent and key independent variables between survey years (2020 and 2023) using the lincom command in STATA [50] to account for the complex survey weights. Several regression specification types were tested to ensure model fit. Ordered logit specifications failed parallel regression assumptions, likely because responses tended to be right-tailed and have a high middle of the distribution (response of – no difference). We tested the multinomial logit, which loses the ordering inherent in our response variable and the generalized ordered logit to relax the parallel regression assumption, but our preferred specifications use ordinary least squares (OLS) with bootstrapped standard errors (100 iterations) to permit within sample correlation (e.g., individuals within regions might have experienced similar COVID-19 regulations). OLS estimators allowed us to keep the ordered nature of dependent variables and bootstrap standard errors as well as run selection bias tests in a straightforward way.

‘Don’t know,’ ‘prefer not to say’ and ‘no answer’ responses were dropped from response variables in model specifications. We tested a set of Heckman selection models [51] for these three responses for each dependent variable to test whether bias was introduced by dropping these responses. Tests confirmed no bias. Therefore, we did not include a sample selection estimator in specifications.

Model goodness of fit was assessed using Wald chi-square values and variance inflation factors (VIF < 10) to ensure lack of multicollinearity. We report stepwise regressions adding explanatory variables in S1–S7 Tables to demonstrate key parameter stability. Additional regressions (not shown here) removing variables (e.g., household income) that could potentially have a recursive relationship with dependent variables were also tested to ensure finding robustness.

## Results

### Study sample characteristics

Table 1 provides descriptive statistics of the weighted sample for each survey year (2020 and 2023) and the total sample. Any significant differences across survey years are denoted with ‘*’ and ‘**’ as appropriate. For the total sample (2020 and 2023), most respondents were from the South Atlantic region of the US (20.0%) followed by Pacific (16.5%) and East North Central (14.5%). A total of 48.6% of participants were men and 51.0% were women. Mean age of participants was 45.3 years. In line with representative quota procedures, there were no statistically significant differences across 2020 and 2023 for region, gender, and age group.

**Table 1:** Study sample descriptive statistics, weighted sample.

	2020	2023	Total
**Observations**	**1,085**	**2,189**	**3,274**
	**Mean (SD)**	**Mean (SD)**	**Mean (SD)**
**Region**			
New England	0.047 (0.211)	0.046 (0.209)	0.046 (0.210)
Middle Atlantic	0.131 (0.338)	0.126 (0.332)	0.128 (0.334)
East North Central	0.146 (0.354)	0.144 (0.351)	0.145 (0.352)
West North Central	0.065 (0.246)	0.064 (0.245)	0.064 (0.245)
South Atlantic	0.198 (0.398)	0.201 (0.401)	0.200 (0.400)
East South Central	0.059 (0.235)	0.058 (0.233)	0.058 (0.234)
West South Central	0.118 (0.323)	0.122 (0.327)	0.120 (0.326)
Mountain	0.072 (0.258)	0.075 (0.264)	0.074 (0.262)
Pacific	0.164 (0.371)	0.165 (0.371)	0.165 (0.371)
**Gender**			
Female	0.512 (0.500)	0.508 (0.500)	0.510 (0.500)
Male	0.488 (0.500)	0.485 (0.500)	0.486 (0.500)
Other gender	0.000 (0.000)	0.003** (0.056)	0.002 (0.046)
Prefer not to say	0.000 (0.000)	0.004** (0.060)	0.002 (0.049)
**Age/ Age group**			
Age	44.88 (15.515)	45.55 (16.096)	45.328 (15.907)
18-24	0.128 (0.334)	0.119 (0.324)	0.122 (0.327)
25-34	0.191 (0.394)	0.196 (0.397)	0.194 (0.396)
35-44	0.182 (0.386)	0.180 (0.384)	0.181 (0.385)
45-54	0.193 (0.395)	0.176 (0.381)	0.182 (0.386)
55-64	0.178 (0.383)	0.171 (0.377)	0.174 (0.379)
65-75	0.127 (0.334)	0.158* (0.365)	0.148 (0.355)
**Household income**			
$0-$24,999	0.109 (0.312)	0.111 (0.314)	0.111 (0.314)
$25,000-$49,999	0.216 (0.412)	0.166* (0.372)	0.182 (0.386)
$50,000-$74,999	0.185 (0.388)	0.163 (0.370)	0.170 (0.376)
$75,000-$99,999	0.191 (0.393)	0.227* (0.419)	0.215 (0.411)
$100,000-$149,999	0.169 (0.375)	0.203* (0.402)	0.192 (0.394)
$150,000-$249,999	0.070 (0.254)	0.069 (0.254)	0.069 (0.254)
$250,000+	0.013 (0.111)	0.020 (0.141)	0.018 (0.132)
Prefer not to say	0.048 (0.213)	0.041 (0.197)	0.043 (0.203)
**Education**			
Below HS	0.017 (0.128)	0.020 (0.141)	0.019 (0.137)
GED or HS diploma	0.134 (0.341)	0.174** (0.379)	0.161 (0.367)
Some college	0.217 (0.412)	0.218 (0.413)	0.217 (0.412)
AS degree	0.121 (0.326)	0.112 (0.315)	0.115 (0.319)
BS degree	0.318 (0.466)	0.280* (0.449)	0.293 (0.455)
MS degree	0.153 (0.360)	0.160 (0.367)	0.158 (0.365)
Professional or Doctorate degree	0.041 (0.198)	0.036 (0.186)	0.037 (0.190)
**Marital status**			
Single, never married	0.289 (0.453)	0.278 (0.448)	0.282 (0.450)
Living with partner	0.073 (0.261)	0.092 (0.289)	0.086 (0.280)
Married	0.506 (0.500)	0.505 (0.500)	0.506 (0.500)
Widowed	0.031 (0.174)	0.030 (0.171)	0.030 (0.172)
Divorced or separated	0.100 (0.301)	0.095 (0.293)	0.097 (0.296)
**Children in household**			
Has children	0.294 (0.456)	0.339* (0.473)	0.324 (0.468)
Does not have children	0.706 (0.456)	0.661* (0.473)	0.676 (0.468)
**Residence rurality** ^**+**^			
Rural	0.127 (0.333)	0.119 (0.324)	0.121 (0.327)
Urban	0.873 (0.333)	0.881 (0.324)	0.879 (0.327)

Notes: + Ipsos supplied core-based statistical area (CBSA) population values for where respondents lived in intervals (nonCBSA, 10,000–99,999, 100,000–249,999; 250,000–499,999; 500,000–999,999; 1,000,000–2,499,999; 2,500,000–4,999,999, 5,000,000+). We defined rural as ‘nonCBSA’ and ‘10,000-99,999’ CBSA population. This may be a slight overestimate as according to Office of Management and Budget and the US Census, ‘rural’ should be below 50,000, however, we were limited by the residence rurality breakdown as supplied.

*p < 0.05, **p < 0.01.

The most common household income group was $75,000-$99,999 (21.5%) with $100,000-$149,999 (19.2%) and $25,000-$49,999 (18.2%) the next most common. Three brackets were statistically different in 2023 versus 2020: $25,000-$49,999, $75,000-$99,999, and $100,000-$149,999. Most participants had at least an associate (AS) degree or higher (60.3%). Education levels only differed between 2020 and 2023 for categories of ‘GED or HS diploma’ (13.4% in 2020, 17.4% in 2023) and ‘BA/BS degree’ (31.8% in 2020 and 28.0% in 2023). Most of the sample was ‘married’ (50.6%) with no statistically significant difference in marital status between 2020 and 2023. In 2023, more people had children <18 years of age in the household than in 2020 (29.4% in 2020, 33.9% in 2023). In the total sample, 67.6% of participants reported no children currently in the household. Most respondents were also from areas designated as urban (87.9%) versus rural (12.1%) with no difference between survey years.

### Changes in healthcare use and health behaviors during and immediately post-COVID-19 pandemic

**Fig 1:**
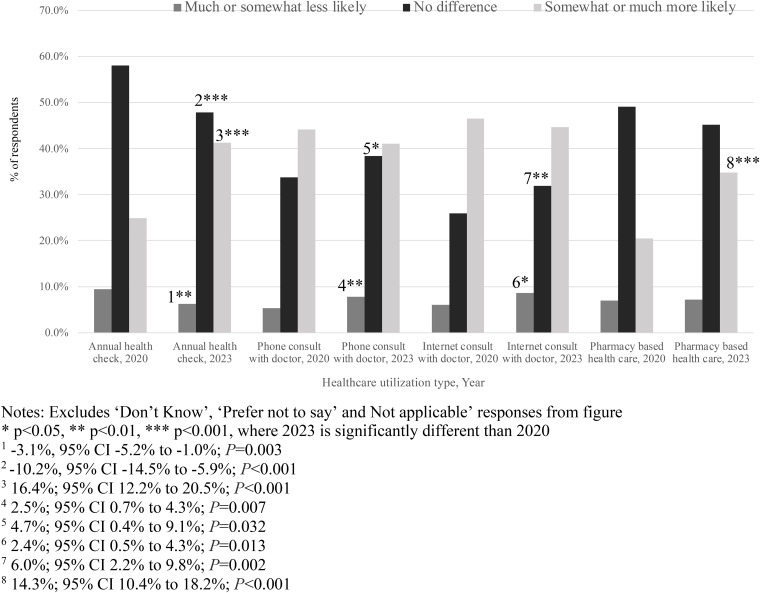
Weighted t-tests of changes in healthcare utilization likelihood since the COVID-19 pandemic, 2020 and 2023.

Fig 1 displays healthcare utilization likelihood in July 2020 and May 2023 (see S8 Table for weighted descriptive statistics). Compared to 2020, respondents in 2023 were more likely to have an annual preventive visit (24.9% in 2020 vs. 41.3% in 2023, 16.4%, 95% CI 12.2% to 20.5%; p < 0.001) and to have sought pharmacy-based healthcare services (20.5% in 2020 vs. 34.7% in 2023, 14.3%, 95% CI 10.4%−18.2%; *P* < 0.001). Respondents in 2023 versus 2020 also had a higher rate of reporting that they were less likely (5.3% in 2020 vs. 7.8% in 2023, 2.5%, 95% CI 0.7% to 4.3%; *P* = 0.007) or had no difference (33.7% in 2020 vs. 38.4% in 2023, 4.7%, 95% CI 0.4% to 9.1%; *P* = 0.032) in likelihood of having a phone consultation with a doctor since the pandemic. Similarly, more respondents in 2023 versus in 2020 expressed no change in likelihood of having a virtual/remote consultation with a healthcare provider since the pandemic (25.9% in 2020 and 31.9% in 2023; 6.0%, 95% CI 2.2% to 9.8%; *P* = 0.002).

For preventive health behaviors, there was an increase in 2023 from 2020 in respondents who reported either being likely (29.0% in 2020 vs. 36.3% in 2023,7.3%, 95% CI 3.1% to 11.5%; *P* = 0.001) or less likely (4.7% in 2020 vs. 9.6% in 2023, 4.9%, 95% CI 3.1% to 6.7%; *P* < 0.001) to get the seasonal influenza vaccine since the pandemic. Respondents’ likelihood to eat healthily increased from 2020 to 2023 since the pandemic (39.8% vs. 50.8%, 11.0%, 95% CI 6.7% to 15.3%; *P* < 0.001). Moreover, there was a sustained increase in participants reporting likelihood of more exercise from 2020 to 2023 compared to before the pandemic (38.9% vs. 46.2%, 7.3%, 95% CI 3.0% to 11.6%; *P* = 0.001).

### Trust in key actors during and immediately post-COVID-19 pandemic

**Fig 2:**
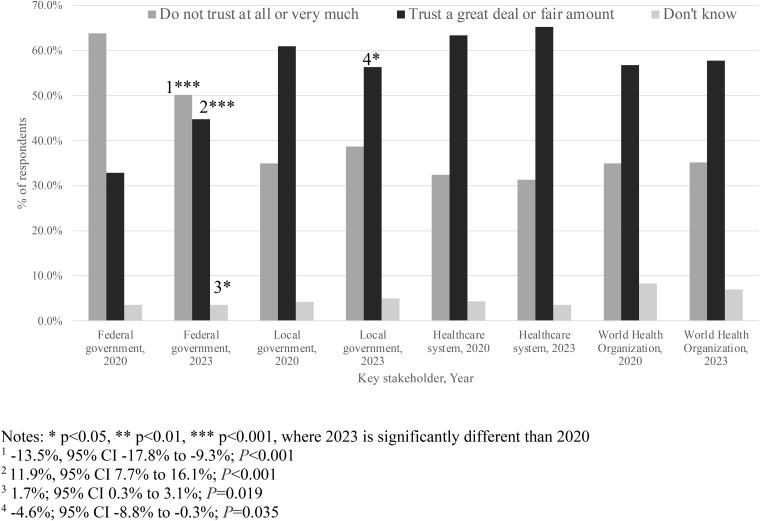
Weighted t-tests of changes in trust in key stakeholders, 2020 and 2023.

The healthcare system was the most trusted among all stakeholders in 2020 (63.3%) and 2023 (65.1%) (S9 Table). Fig 2 displays these results graphically. The federal government was the least trusted entity at both time points, although mistrust declined from 2020 to 2023 (63.8% versus 50.2%, −13.5%, 95% CI −17.8% to −9.3%; *P* < 0.001). Related, trust in the federal government increased from 2020 to 2023 (32.8% versus 44.7%; 11.9%, 95% CI 7.7% to 16.1%; *P* < 0.001). However, trust in local government decreased from 2020 to 2023 (60.9% vs. 56.3%, −4.6%, 95% CI −8.8% to 0.3%; *P* = 0.04). There were no significant differences between the two time periods for trust in the WHO (56.7% in 2020, 57.7% in 2023, 1.0%, 95% CI −3.3% to 5.3%; *P* = 0.65).

### Household finances during and immediately post-COVID-19 pandemic

Household finances had significant changes from 2020 to 2023 (S9 Table). In 2023, the response with the highest percentage of respondents was household financial status being much worse or a little worse than before the pandemic (33.3% in 2020 vs. 41.5% in 2023, 8.2%, 95% CI 4.3% to 12.1%; *P* < 0.001). This was a significant increase from 2020. However, also, there was an increase in respondents reporting that their finances were much better or a little better than before the pandemic in 2023 versus 2020 (16.4% in 2020 vs. 24.7% in 2023, 8.3%, 95% CI 4.2% to 12.4%; *P* < 0.001). This increase from 2020 was also significant. Therefore, more respondents reporting moving to either a better or worse household financial position in 2023 than in 2020. Related, in 2020, the most common response was ‘no difference’ in household finances than before the pandemic (45.7%) but this number significantly declined (28.0%) in 2023 (−17.7%, 95% CI −21.9% to −13.5%; *P* < 0.001).

### Trust, household finances and healthcare use

Table 2 presents regression results for associations between trust, household finances, and healthcare use likelihood. Models shown are our preferred specifications after stepwise selection (stepwise results in S1–S7 Tables).

**Table 2:** Ordinary least squares regressions or healthcare utilization likelihood since the COVID-19 pandemic.

VARIABLES	Annual Health Check	Phone Consultation with Doctor	Virtual/ Remote Consultation with Doctor	Pharmacy-Based Healthcare
	Coef. (Std. Err.)	Coef. (Std. Err.)	Coef. (Std. Err.)	Coef. (Std. Err.)
**Trust in federal government** **(ref = Trust a great deal)**				
*Trust a fair amount*	−0.099	−0.054	0.071	−0.177*
	(0.080)	(0.075)	(0.083)	(0.073)
*Do not trust very much*	−0.147	−0.053	−0.020	−0.304***
	(0.086)	(0.077)	(0.097)	(0.076)
*Do not trust at all*	−0.247**	0.013	0.104	−0.321***
	(0.085)	(0.084)	(0.099)	(0.074)
**Trust in local government** **(ref = Trust a great deal)**				
*Trust a fair amount*	−0.028	−0.028	−0.079	−0.031
	(0.060)	(0.067)	(0.065)	(0.060)
*Do not trust very much*	−0.035	−0.100	−0.008	−0.001
	(0.067)	(0.080)	(0.076)	(0.071)
*Do not trust at all*	0.039	−0.222*	−0.203*	−0.106
	(0.079)	(0.093)	(0.097)	(0.083)
**Trust in the healthcare system** **(ref = Trust a great deal)**				
*Trust a fair amount*	−0.131*	−0.236***	−0.267***	−0.184***
	(0.052)	(0.052)	(0.055)	(0.052)
*Do not trust very much*	−0.277***	−0.265***	−0.274***	−0.272***
	(0.067)	(0.068)	(0.073)	(0.062)
*Do not trust at all*	−0.477***	−0.491***	−0.423***	−0.453***
	(0.090)	(0.092)	(0.098)	(0.083)
**Trust in the World Health Organization** **(ref = Trust a great deal)**				
*Trust a fair amount*	−0.083	−0.108*	−0.069	−0.057
	(0.056)	(0.054)	(0.061)	(0.055)
*Do not trust very much*	−0.207**	−0.221***	−0.227**	−0.186**
	(0.067)	(0.059)	(0.072)	(0.061)
*Do not trust at all*	−0.144*	−0.249***	−0.283***	−0.208**
	(0.073)	(0.071)	(0.081)	(0.072)
**Household finances (ref = Much better)**				
*A little better*	−0.144	−0.057	−0.025	−0.082
	(0.092)	(0.103)	(0.097)	(0.092)
*A little worse*	−0.169*	−0.072	−0.051	−0.185*
	(0.081)	(0.088)	(0.085)	(0.085)
*Much worse*	−0.215*	0.010	−0.060	−0.127
	(0.085)	(0.097)	(0.097)	(0.085)
*No difference*	−0.338***	−0.168	−0.155*	−0.277**
	(0.088)	(0.095)	(0.076)	(0.089)
**Year (ref = 2020)**				
*2023*	0.283***	−0.125**	−0.094*	0.138***
	(0.035)	(0.040)	(0.044)	(0.040)
**Self-reported physical health** **(ref = Very good or good)**				
*Fair*	0.026	0.073	0.045	0.0143
	(0.049)	(0.048)	(0.059)	(0.051)
*Poor or very poor*	0.005	0.166	0.126	−0.016
	(0.097)	(0.091)	(0.095)	(0.081)
**Self-reported mental health** **(ref = Very good or good)**				
*Fair*	−0.033	0.074	0.065	−0.018
	(0.044)	(0.043)	(0.052)	(0.044)
*Poor or very poor*	−0.048	0.043	0.063	0.045
	(0.071)	(0.065)	(0.077)	(0.060)
Constant	3.675***	4.451***	3.965***	3.795***
	(0.183)	(0.181)	(0.168)	(0.172)
Wald x2 (p-value)	1705.11 (0.000)	562.34 (0.000)	893.12 (0.000)	1463.06 (0.000)
Mean VIF	2.69	2.69	2.70	2.72
R^2^	0.119	0.093	0.088	0.126
Observations	3039	2757	2670	2681

Standard errors in parentheses.

*p < 0.05, **p < 0.01, ***p < 0.001.

Models include gender, age, household income, education, marital status, children in household, region, residence rurality.

Trust in the healthcare system was positively related to healthcare utilization. Of all stakeholders elicited in the survey, trust in the healthcare system had the greatest effect sizes on outcomes. For example, extreme mistrust in the US healthcare system was associated with lower likelihood of utilization of all queried healthcare services since the pandemic (−47.7% [annual health check], −49.1% [phone consultation], −42.3% [virtual/remote consultation] and −45.3% [pharmacy-based healthcare]).

In addition, mistrust in the WHO was associated with lower likelihood of using all four healthcare services (see Table 2). Mistrust in the federal government was strongly negatively associated with individuals’ use of pharmacy-based care, with a marked decrease in use when participants felt less than ‘great trust in the national [federal] government’ (ranging from-17.7% with ‘trust a fair amount’ to −32.1% with ‘do not trust at all’).

Worse finances (‘a little worse’, −16.9%; ‘much worse’ −21.5%) and ‘no difference’ (−33.8%) were associated with lower likelihood of an annual health check when compared to individuals who described their finances as ‘much better.’ ‘No difference’ in household finances (versus finances being ‘much better’) was associated with lower likelihood of virtual/remote consultation (−15.5%) and pharmacy-based services (−27.7%). Changes in household finances had no association with phone consultation likelihood and a non-linear association with use of pharmacy-based services.

### Trust, household finances and preventive behaviors

Mistrust in the healthcare system and WHO were major contributing factors for reduced likelihood of getting a seasonal influenza vaccine since the pandemic (Table 3). Changes in household finances and self-reported mental health were not associated with likelihood of getting a seasonal influenza vaccination. However, being in ‘fair’ physical health relative to ‘very good’ or ‘good’ was positively associated (12.5%) with likelihood of vaccination.

**Table 3:** Ordinary least squares regression for preventive health behaviors likelihood since the COVID-19 pandemic.

VARIABLES	Seasonal Influenza Vaccination	Eat Healthily	Exercise
	Coef. (Std. Err.)	Coef. (Std. Err.)	Coef. (Std. Err.)
**Trust in federal government (ref = Trust a great deal)**			
*Trust a fair amount*	−0.066	−0.063	−0.222**
	(0.082)	(0.067)	(0.075)
*Do not trust very much*	−0.145	−0.154*	−0.306***
	(0.096)	(0.070)	(0.084)
*Do not trust at all*	−0.174	−0.096	−0.282**
	(0.100)	(0.082)	(0.089)
**Trust in local government (ref = Trust a great deal)**			
*Trust a fair amount*	−0.039	−0.036	−0.013
	(0.062)	(0.054)	(0.055)
*Do not trust very much*	−0.109	−0.032	−0.048
	(0.074)	(0.064)	(0.066)
*Do not trust at all*	−0.046	0.004	−0.066
	(0.099)	(0.067)	(0.080)
**Trust in the healthcare system (ref = Trust a great deal)**			
*Trust a fair amount*	−0.166**	−0.093	−0.026
	(0.055)	(0.051)	(0.050)
*Do not trust very much*	−0.271***	−0.251***	−0.077
	(0.071)	(0.061)	(0.064)
*Do not trust at all*	−0.510***	−0.189*	0.097
	(0.108)	(0.074)	(0.089)
**Trust in the World Health Organization** **(ref = Trust a great deal)**			
*Trust a fair amount*	−0.122*	−0.116*	−0.086
	(0.059)	(0.051)	(0.052)
*Do not trust very much*	−0.374***	−0.163*	−0.143*
	(0.077)	(0.065)	(0.063)
*Do not trust at all*	−0.515***	−0.174**	−0.138*
	(0.080)	(0.067)	(0.066)
**Household finances** **(ref = Much better)**			
*A little better*	−0.078	−0.181*	−0.163
	(0.096)	(0.088)	(0.086)
*A little worse*	−0.060	−0.164*	−0.233**
	(0.106)	(0.082)	(0.076)
*Much worse*	−0.134	−0.164*	−0.206**
	(0.112)	(0.082)	(0.070)
*No difference*	−0.194	−0.318***	−0.307***
	(0.102)	(0.078)	(0.072)
**Year (ref = 2020)**			
*2023*	0.012	0.147***	0.135***
	(0.042)	(0.032)	(0.036)
**Self-reported physical health (ref = Very good or good)**			
*Fair*	0.125**	−0.086	−0.167***
	(0.047)	(0.048)	(0.044)
*Poor or very poor*	0.005	−0.086	−0.174*
	(0.098)	(0.090)	(0.088)
**Self-reported mental health** **(ref = Very good or good)**			
*Fair*	−0.007	−0.076	−0.092*
	(0.052)	(0.043)	(0.046)
*Poor or very poor*	−0.061	−0.217***	−0.174**
	(0.075)	(0.065)	(0.060)
_cons	3.905***	3.886***	3.640***
	(0.204)	(0.180)	(0.188)
Wald x2 (p-value)	1207.26 (0.000)	778.57 (0.000)	868.69 (0.000)
Mean VIF	2.66	2.70	2.69
R^2^	0.119	0.094	0.099
Observations	2781	3125	3025

Standard errors in parentheses.

*p < 0.05, **p < 0.01, ***p < 0.001.

Models include gender, age, household income, education, marital status, children in household, region, residence rurality.

Mistrust in the federal government (−15.4% ‘do not trust very much’ versus ‘trust a great deal’) and the WHO (−17.4% ‘do not trust at all’ versus ‘trust a great deal’) were associated with lower likelihood of eating healthily. Mistrust in healthcare systems was also associated with lower likelihood of eating healthily (−25.1% ‘do not trust very much’ and –18.9% ‘do not trust at all versus ‘trust a great deal’).

Reporting household finances as being ‘much better’ since the pandemic was associated with higher likelihood of eating healthy (−16.4% ‘little worse,’ −16.4% ‘much worse’ and −18.1% ‘a little better’ versus ‘much better’). Responding to the survey in 2023 versus 2020 was also associated with higher likelihood of eating healthily (14.7%). Mental health but not physical health was associated with likelihood of eating healthily with respondents describing their mental health as ‘poor or very poor’ relative to ‘very good or good’ being less likely to eat healthily (−21.7%).

As with eating healthily, likelihood of exercising was greater if respondents trusted the federal government (−28.2% ‘do not trust at all’ vs. ‘trust a great deal’) or the WHO (-13.8% “do not trust at all’ vs. ‘trust a great deal’), had improved finances over the pandemic (−23.3%, ‘a little worse’ and −20.6%, ‘much worse’ vs. ‘much better’), were responding to the survey in 2023 versus 2020 (13.5%) and self-reported ‘good’ or ‘very good’ mental health (−17.4% for both ‘poor’ or ‘very poor’). Self-reported physical health status was important for exercise but not eating healthily.

## Discussion

This study employed two nationally representative surveys of US residents to investigate associations between trust in key stakeholders, changes in household finances, and healthcare utilization and preventive health behaviors during and immediately post-COVID-19 pandemic. Overall, US residents reported greater likelihoods of engaging in several types of healthcare and preventive behaviors post-pandemic, with both trust and household finances being associated with behaviors in unique ways.

Participants showed increased engagement in annual health checks and in pharmacy-based healthcare from 2020 to 2023. Both pharmacy-based care and annual health checks are in-person options, suggesting a desire post-pandemic to engage in in-person services alongside of new care modalities. Coupled with evidence that adherence to recommended screenings/tests and referrals may be lower in telehealth visits versus in-person [52], these results highlight the importance of offering patients both in-person and virtual modalities based on patient preference, visit reason and access barriers. Existing research demonstrates that patients in the US find pharmacy-based care for activities such as colorectal cancer screening [53] and vaccination [54] acceptable, thus supporting findings that this newly expanded modality of care shows promise for future preventive care and chronic disease management in community-based settings.

Vaccine hesitancy related to COVID-19 vaccines may very well spillover into seasonal influenza vaccination [17,55] and intentions to vaccinate for either virus may have similar underlying reasons [44]. The current study’s findings support that uptake of seasonal influenza vaccination have become more bifurcated from 2020 to 2023.

While research from early in the pandemic suggested that the pandemic had negative impacts on healthy eating [15] and exercise [16] our findings provide evidence of a ‘bounce-back’ in terms of growth in the percentage of individuals reporting eating more healthily in 2023. These changes may reflect easier attainment of healthy foods compared to early in the pandemic due to factors such as supply chain shortages. Previous research has documented increased unhealthy food and beverage consumption in early pandemic waves for US residents, particularly during periods of home confinement [56,57].

When considering trust in key institutions, trust among US residents continued to be high in the healthcare system relative to other institutional actors. This supports previous findings that trust in the healthcare system was high during COVID-19 and is consistent with evidence from Europe that the pandemic drove trust in healthcare institutions [58]. Trust also increased over time in the federal government which could be related to how changes in Presidential and US House of Representatives leadership over our study period affected our sample’s trust in the federal government.

Upon examining the relationship between trust and healthcare utilization and preventive health behaviors, two key findings stand out. First, trust in the healthcare system was associated with all healthcare use types, as well as uptake of seasonal influenza immunization, supporting previous evidence about the critical role of trust in delivery of healthcare services [59] including vaccination [60]. Second and interestingly, trust in the WHO was found to be associated with *all* types of healthcare utilization and preventive behaviors. The WHO was the only stakeholder of those tested (healthcare system, local government, federal government) where this was the case. This is particularly notable in a US sample where the WHO’s handling of the pandemic has been scrutinized. However, according to social media data, many US residents relied on the WHO as an information source about the COVID-19 pandemic, along with the CDC, news outlets and the White House [61]. Our sample was only slightly lower in trusting the WHO’s ability to handle the pandemic (35.0% in 2020) than another nationally representative survey in 2020 (38.5%) [62].

As for changes in household finances, among healthcare utilization and health behaviors examined, they were most strongly associated with self-reports of eating healthily and exercising, as well as receiving annual health checks. As eating healthily costs more than eating less nutrient-dense and more processed food, this finding has implications for how economic shocks may impact health behaviors. Notably, inflation, particularly food-related inflation, may have direct impacts on healthy eating intentions and how people perceive their household financial status is an important component of understanding inflation’s impact on consumption behaviors. This is an important area for future research given annual inflation rates since the COVID-19 pandemic. Similarly, and likely also related to inflationary effects, getting exercise was also associated with changes in household finances. Exercise has opportunity costs in terms of time and other aspects (e.g., gym membership, travel to exercise). Household finances may be a key barrier to residents realizing desires to eat healthily and exercise. These results are underlined by the fact that models control for household income, which isolates the specific effect of changes in household finance during the pandemic period.

Finally, self-reported health status was important for healthier eating and increased exercise amongst respondents. Individuals who reported poorer mental health were shown to be at greater risk of not eating healthy or exercising more since the pandemic. This is a worrying recursive cycle given the known mental health benefits of good diet and exercise.

### Limitations

These findings must be interpreted within existing limitations. Our study is limited by the repeated cross-sectional survey design. This precludes following the same individuals over time. However, the nationally representative nature of the survey attenuates some of this by offering responses reflective of the US population according to dimensions used for representativeness. The sample is, however, limited to individuals with internet access (either computer or via smartphone) as Ipsos does not provide devices for this panel to complete surveys. According to Pew Research, 95% of Americans used the internet in 2023 with 80% reporting home broadband and an additional 15% relying solely on a smartphone or internet access [63]. This high percentage of internet access supports the generalizability of our findings. While individuals with lower education and income were more likely to not have internet access [63], Ipsos provided weights to account for the non-online portion of the US population that specifically account for, amongst other characteristics, working status and household income.

Related, non-responders who were replaced by other individuals from their quota sample may introduce some bias in the results. Since they were replaced with individuals from their quota sample, this limited any bias in demographics but if individuals chose not to take the survey because of the questions or topics covered, this could create bias for reasons such as not wanting to report or reflect on negative experiences or lack of healthcare use. This would result in over-reporting survey responses such as having the same or more healthcare utilization or preventive health behaviors, increased trust in stakeholders and stable or improved household finances. However, individuals may have chosen not to respond for other reasons such as being busy at the time the prompt arrived. If employment or childcare responsibilities were the reasons for being busy, we were able to mitigate any bias caused by this through controlling for whether there were children in the household. Income only partially controlled for being busy through employment since income may not necessarily be associated with employment status (e.g., retirement).

Participants were asked to consider their health behaviors and healthcare utilization since the pandemic started. Recall effects may be a threat to response validity, namely for the second survey as participants were asked to consider a time approximately three years prior. The salience of the COVID-19 pandemic and how it fundamentally changed people’s lives somewhat attenuates recall bias concerns. Related, respondents self-reported healthcare use (e.g., preventive visits, vaccination) that could not be confirmed by clinical data. This study attempted to mitigate this risk by not asking specific questions about how many visits and referencing a salient period of time, the COVID-19 pandemic to help with recall time frame [64].

Also, responses were subject to social desirability bias. Social desirability bias would cause underreporting of responses that individuals perceived as less positive about themselves and overreporting of responses perceived to be more positive. Questions asking about healthy eating and physical activity would be particularly prone to such a bias, leading to overestimates of healthy behaviors. Our survey aimed to mitigate this risk through question design by offering respondents the option of ‘no change’ so that their state (health or unhealthy eating, more or less exercise) would not be revealed. Respondents also could select ‘don’t know and ‘prefer not to say’ options. Similarly, respondents may have underreported negative changes in household finances and/or overreported household finances being better. Again, respondents had neutral and ‘don’t know’ and ‘prefer not to say’ options to attempt to mitigate this bias.

Finally, this study only included variables where we had responses for both survey years (2020, 2023), therefore our models might not incorporate all factors potentially associated with likelihood of healthcare use and preventive behavior. The number of survey questions asked was also limited by study budget and scope. Thus, not all parameters that we might include in specifications were asked or available for both years or at all (e.g., partisanship/ party affiliation, presidential voting record in the 2016 and 2020 elections, perceptions about the US economy, insurance status, having a regular doctor, other preventive behaviors). In addition, while respondents were asked generally about their trust in the federal government in both survey years, they were not specifically queried about entities of the government such as the presidential administration (only asked in 2023) or the Centers for Disease Control and Prevention (CDC) (not asked in either year). Future studies that investigate attitudes and perceptions of specific federal entities and their relationship with health behaviors, including COVID-19 vaccination is warranted.

### Implications for practice and policy

We observed that the COVID-19 pandemic brought more intentions to engage in several health seeking behaviors. Moreover, in-person opportunities for healthcare in the form of annual health checks and pharmacy-based service continued to see increases in those who planned to utilize those services since the pandemic. This has important implications both in terms of continuing to help individuals re-engage in their healthcare post-pandemic and identify ways to optimize care via in-person and virtual services. We also observed increased intentions to engage in healthy behaviors in the forms of healthy eating and exercise that continued to grow in the post-pandemic period. The picture with influenza vaccination is more complicated with US residents appearing increasingly polarized since the COVID-19 pandemic in their vaccination intentions for this annual vaccine.

Taken together, we see evidence that COVID-19 yielded shifts in US residents’ beliefs about various health behaviors. Insurers, employers, federal and state payers and healthcare systems can use these results to spur engagement in helping US residents realize these intentions into behaviors. Making annual preventive health checks easier to access, reimbursing or providing gym memberships or population-specific exercise classes or walking groups, offering healthy eating and cooking classes as well as options to do so in the workplace and community are all ways these actors can facilitate health behavior change for US residents emerging from the pandemic with positive health goals. With household finances as a barrier, specifically to exercise and healthy eating, these actors can help, and all will benefit.

Trust continues to be high in healthcare systems during the immediate post-COVID-19 pandemic period. Still, those lacking trust in healthcare systems had limited uptake of healthcare services and seasonal influenza immunization. Maintaining trust in healthcare systems through ensuring patient privacy [65], collaborating with community-based partners and trusted members of the community [66] and strengthening patient-provider relationships along with patient trust in care teams [67] are key to next steps. These activities to support trust in the health system are key to promoting positive health behaviors and adequate healthcare utilization that will help address the wide-ranging health and mental health needs existing in the current post-pandemic era or in future public health emergencies. Efforts to maintain and increase individuals’ trust in healthcare providers and systems are critical—particularly within the context of a global pandemic when use of certain healthcare services such as vaccination may play a fundamental role in protecting individual and public health, as well as ending outbreaks. Trust in healthcare systems is also likely to be important for promoting re-engagement in care following periods of care disengagement or disruptions in health services due to external factors (e.g., pandemics, natural disasters).

## Supporting information

S1 File Study survey. Questions asked on behalf of the research team by Ipsos Online Global Omnibus in July 10–14, 2020 and May 24–29, 2023.
(PDF)

S1 Table Stepwise ordinary least squares regression for annual health check. (PDF)

S2 Table Stepwise ordinary least squares regression for phone consultation with doctor. (PDF)

S3 Table Stepwise ordinary least squares regression for virtual consultation with doctor. (PDF)

S4 Table Stepwise ordinary least squares regression for pharmacy-based healthcare. (PDF)

S5 Table Stepwise ordinary least squares regression for seasonal influenza vaccine. (PDF)

S6 Table Stepwise ordinary least squares regression for eating healthily. (PDF)

S7 Table Stepwise ordinary least squares regression for exercising. (PDF)

S8 Table Weighted descriptive statistics for dependent variables, 2020 and 2023. (PDF)

S9 Table Weighted descriptive statistics for key study independent variables, 2020 and 2023. (PDF)

## References

[1] Behavioural economics and policy for pandemics. Insights from responses to COVID-19. Cambridge, UK: Cambridge University Press; 2024.

[2] Liu Y, Zhang Y, Chatterjee S. Financial hardship and depression experienced by pre-retirees during the COVID-19 pandemic: the mitigating role of stimulus payments. Appl Econ Lett. 2023;30(3):391–6.

[3] Hair NL, Urban C. Association of severe COVID-19 and persistent COVID-19 symptoms with economic hardship among US families. JAMA Netw Open. 2023;6(12):e2347318. doi: 10.1001/jamanetworkopen.2023.47318 38085541 PMC10716716

[4] Chetty R, Friedman JN, Stepner M, Opportunity Insights Team. The economic impacts of covid-19: evidence from a new public database built using private sector data. Q J Econ. 2024;139(2):829–89. doi: 10.1093/qje/qjad048 38911676 PMC11189622

[5] SteelFisher GK, Findling MG, Caporello HL, Lubell KM, Vidoloff Melville KG, Lane L, et al. Trust in US federal, state, and local public health agencies during COVID-19: responses and policy implications. Health Aff. 2023;42(3):328–37. 10.1377/hlthaff.2022.01204 PMC11318038 36877902

[6] Siegrist M, Bearth A. Worldviews, trust, and risk perceptions shape public acceptance of COVID-19 public health measures. Proc Natl Acad Sci U S A. 2021;118(24). 10.1073/pnas.2100411118 PMC8214671 34045360

[7] Slovic P. Trust, emotion, sex, politics, and science: surveying the risk-assessment battlefield. Risk Anal. 1999;19(4):689–701. doi: 10.1023/a:1007041821623 10765431

[8] Baker DW. Trust in health care in the time of COVID-19. JAMA. 2020;324(23):2373–5. doi: 10.1001/jama.2020.23343 33320208

[9] Moynihan R, Sanders S, Michaleff ZA, Scott AM, Clark J, To EJ, et al. Impact of COVID-19 pandemic on utilisation of healthcare services: a systematic review. BMJ Open. 2021;11(3):e045343. doi: 10.1136/bmjopen-2020-045343 33727273 PMC7969768

[10] Whaley CM, Pera MF, Cantor J, Chang J, Velasco J, Hagg HK, et al. Changes in health services use among commercially insured US populations during the COVID-19 pandemic. JAMA Netw Open. 2020;3(11):e2024984. 10.1001/jamanetworkopen.2020.24984 PMC7645698 33151319

[11] Klepser DG, Klepser NS, Adams JL, Adams AJ, Klepser ME. The impact of the COVID-19 pandemic on addressing common barriers to pharmacy-based point-of-care testing. Expert Rev Mol Diagn. 2021;21(8):751–5. doi: 10.1080/14737159.2021.1944105 34130575

[12] Hung P, Yu J, Harrison SE, Liu J, Promiti A, Odahowski C, et al. Racial and ethnic and rural variations in the use of hybrid prenatal care in the US. JAMA Netw Open. 2024;7(12):e2449243. doi: 10.1001/jamanetworkopen.2024.49243 39641928 PMC11624583

[13] Friedman AB, Gervasi S, Song H, Bond AM, Chen AT, Bergman A, et al. Telemedicine catches on: changes in the utilization of telemedicine services during the COVID-19 pandemic. Am J Manag Care. 2022;28(1):e1–6. doi: 10.37765/ajmc.2022.88771 35049260

[14] Mehrotra A, Bhatia RS, Snoswell CL. Paying for telemedicine after the pandemic. JAMA. 2021;325(5):431–2. 33528545 10.1001/jama.2020.25706 PMC9320940

[15] Robinson E, Boyland E, Chisholm A, Harrold J, Maloney NG, Marty L, et al. Obesity, eating behavior and physical activity during COVID-19 lockdown: a study of UK adults. Appetite. 2021;156:104853. doi: 10.1016/j.appet.2020.104853 33038479 PMC7540284

[16] Tison GH, Avram R, Kuhar P, Abreau S, Marcus GM, Pletcher MJ. Worldwide effect of COVID-19 on physical activity: a descriptive study. Ann Intern Med. 2020;173(9):767–70. 32598162 10.7326/M20-2665 PMC7384265

[17] Leuchter RK, Jackson NJ, Mafi JN, Sarkisian CA. Association between Covid-19 vaccination and influenza vaccination rates. N Engl J Med. 2022;386(26):2531–2. 35704429 10.1056/NEJMc2204560 PMC9258773

[18] Birkhäuer J, Gaab J, Kossowsky J, Hasler S, Krummenacher P, Werner C, et al. Trust in the health care professional and health outcome: a meta-analysis. PLoS One. 2017;12(2):e0170988. doi: 10.1371/journal.pone.0170988 28170443 PMC5295692

[19] Smith LE, Amlôt R, Weinman J, Yiend J, Rubin GJ. A systematic review of factors affecting vaccine uptake in young children. Vaccine. 2017;35(45):6059–69. doi: 10.1016/j.vaccine.2017.09.046 28974409

[20] Callaghan T, Moghtaderi A, Lueck JA, Hotez P, Strych U, Dor A, et al. Correlates and disparities of intention to vaccinate against COVID-19. Soc Sci Med. 2021;272:113638. doi: 10.1016/j.socscimed.2020.113638 33414032 PMC7834845

[21] Marie Reinhart A, Tian Y, Lilly AE. The role of trust in COVID-19 vaccine hesitancy and acceptance among Black and White Americans. Vaccine. 2022;40(50):7247–54. doi: 10.1016/j.vaccine.2022.10.067 36333223 PMC9618447

[22] Lamuda PA, Azar A, Taylor BG, Balawajder EF, Pollack HA, Schneider JA. Latent class analysis of medical mistrust and COVID-19 vaccine hesitancy among adults in the United States just prior to FDA emergency use authorization. Vaccine. 2023;41(16):2671–9. doi: 10.1016/j.vaccine.2023.03.016 36933985 PMC10008805

[23] Lee Y-Y, Lin JL. How much does trust really matter? A study of the longitudinal effects of trust and decision-making preferences on diabetic patient outcomes. Patient Educ Couns. 2011;85(3):406–12. doi: 10.1016/j.pec.2010.12.005 21269794

[24] Hall MA, Dugan E, Zheng B, Mishra AK. Trust in physicians and medical institutions: what is it, can it be measured, and does it matter? Milbank Q. 2001;79(4):613–39, v. doi: 10.1111/1468-0009.00223 11789119 PMC2751209

[25] Ramaswamy A, Yu M, Drangsholt S, Ng E, Culligan PJ, Schlegel PN, et al. Patient satisfaction with telemedicine during the COVID-19 pandemic: retrospective cohort study. J Med Internet Res. 2020;22(9):e20786. doi: 10.2196/20786 32810841 PMC7511224

[26] Liu JYW, Sorwar G, Rahman MS, Hoque MR. The role of trust and habit in the adoption of mHealth by older adults in Hong Kong: a healthcare technology service acceptance (HTSA) model. BMC Geriatr. 2023;23(1):73. 36737712 10.1186/s12877-023-03779-4 PMC9898708

[27] DeMartino JK, Swallow E, Goldschmidt D, Yang K, Viola M, Radtke T. Direct health care costs associated with COVID-19 in the United States. J Manag Care Spec Pharm. 2022;28(9):936–47. 35722829 10.18553/jmcp.2022.22050 PMC12101566

[28] Richards F, Kodjamanova P, Chen X, Li N, Atanasov P, Bennetts L. Economic burden of COVID-19: a systematic review. Clinicoecon Outcomes Res. 2022;14:293–307. 35509962 10.2147/CEOR.S338225 PMC9060810

[29] Graves JA, Baig K, Buntin M. The financial effects and consequences of COVID-19: a gathering storm. JAMA. 2021;326(19):1909–10. doi: 10.1001/jama.2021.18863 34714325

[30] Lim K, Zabek M. Women’s labor force exits during COVID-19: differences by motherhood, race, and ethnicity. J Fam Econ Iss. 2024;45(3):504–27.

[31] Adams-Prassl A, Boneva T, Golin M, Rauh C. Inequality in the impact of the coronavirus shock: evidence from real time surveys. J Public Econ. 2020;189:104245.

[32] Long T, Zhang K, Chen Y, Wu C. Trends in diet quality among older US adults from 2001 to 2018. JAMA Netw Open. 2022;5(3):e221880. doi: 10.1001/jamanetworkopen.2022.1880 35275167 PMC8917422

[33] McCullough ML, Chantaprasopsuk S, Islami F, Rees-Punia E, Um CY, Wang Y, et al. Association of socioeconomic and geographic factors with diet quality in US adults. JAMA Netw Open. 2022;5(6):e2216406. doi: 10.1001/jamanetworkopen.2022.16406 35679041 PMC9185183

[34] Armstrong S, Wong CA, Perrin E, Page S, Sibley L, Skinner A. Association of physical activity with income, race/ethnicity, and sex among adolescents and young adults in the United States: findings from the National Health and Nutrition Examination Survey, 2007-2016. JAMA Pediatr. 2018;172(8):732–40. 29889945 10.1001/jamapediatrics.2018.1273 PMC6142913

[35] Elgaddal N KE, Reuben C. Physical activity among adults aged 18 and over: United States, 2020. Hyattsville (MD): NCHS Data Brief; 2022. Contract No.: no 443. 36043905

[36] Shuval K, Li Q, Gabriel KP, Tchernis R. Income, physical activity, sedentary behavior, and the “weekend warrior” among U.S. adults. Prev Med. 2017;103:91–7. doi: 10.1016/j.ypmed.2017.07.033 28802654

[37] Andersen R, Newman JF. Societal and individual determinants of medical care utilization in the United States. Milbank Q. 2005;83(4). 4198894

[38] Yakusheva O, van den Broek-Altenburg E, Brekke G, Atherly A. Lives saved and lost in the first six month of the US COVID-19 pandemic: a retrospective cost-benefit analysis. PLoS One. 2022;17(1):e0261759. doi: 10.1371/journal.pone.0261759 35061722 PMC8782469

[39] Statement on the fifteenth meeting of the IHR (2005) Emergency Committee on the COVID-19 pandemic [press release]. World Health Organization; 2023.

[40] COVID-19 Public Health Emergency (PHE) [press release]. US Department of Health and Human Services; 2023.

[41] Ipsos. ESOMAR: Ipsos answers to ESOMAR questions for users and buyers of online samples; 2022.

[42] Costa-Font J, Rudisill C, Harrison S, Salmasi L. The social value of a SARS-CoV-2 vaccine: willingness to pay estimates from four western countries. Health Econ. 2023;32(8):1818–35. doi: 10.1002/hec.4690 37151130

[43] Rudisill C, Harrison S. Trust and the COVID-19 pandemic. In: Costa-Font J, Gallizzi M, editors. Behavioral economics and policy for pandemics: insights from responses to COVID-19. Cambridge, UK: Cambridge University Press; 2024. p. 378–400.

[44] Chupak AL, Rudisill C, Harrison S, Linvill K, Costa-Font J, Hung P, et al. Impact of perceived neighborhood social cohesion on vaccination intentions in the post-pandemic era. Prev Med. 2024;189:108158. doi: 10.1016/j.ypmed.2024.108158 39481611 PMC11917178

[45] Hamilton CM, Strader LC, Pratt JG, Maiese D, Hendershot T, Kwok RK, et al. The PhenX Toolkit: get the most from your measures. Am J Epidemiol. 2011;174(3):253–60. doi: 10.1093/aje/kwr193 21749974 PMC3141081

[46] CDC COVID-19 Community Survey Question Bank [Internet]. National Library of Medicine; 2020. Available from: https://cde.nlm.nih.gov/formView?tinyId=Kcceysolt

[47] Gallup. Trust in Government; 2024 [cited 2024 Nov 20]. Available from: https://news.gallup.com/poll/5392/trust-government.aspx

[48] Understanding American Survey COVID-19 Panel UA230 Survey Questionnaire National. USC Dornsife Center for Economic and Social Research. Available from: https://uasdata.usc.edu/index.php

[49] Hays RD, Bjorner JB, Revicki DA, Spritzer KL, Cella D. Development of physical and mental health summary scores from the patient-reported outcomes measurement information system (PROMIS) global items. Qual Life Res. 2009;18(7):873–80. doi: 10.1007/s11136-009-9496-9 19543809 PMC2724630

[50] StataCorp. Stata statistical software: release 16. College Station (TX): StataCorp LLC; 2019.

[51] Heckman JJ. Sample selection bias as a specification error. Econometrica. 1979;47(1):153–61.

[52] Zhong A, Amat MJ, Anderson TS, Shafiq U, Sternberg SB, Salant T, et al. Completion of recommended tests and referrals in telehealth vs in-person visits. JAMA Netw Open. 2023;6(11):e2343417. doi: 10.1001/jamanetworkopen.2023.43417 37966837 PMC10652149

[53] Ferrari RM, Atkins DL, Wangen M, Rohweder CL, Waters AR, Correa S. Patient perspectives on a proposed pharmacy-based colorectal cancer screening program. Transl Behav Med. 2023;13(12):909–18. 37756664 10.1093/tbm/ibad057 PMC10724111

[54] Burson RC, Buttenheim AM, Armstrong A, Feemster KA. Community pharmacies as sites of adult vaccination: a systematic review. Hum Vaccin Immunother. 2016;12(12):3146–59. doi: 10.1080/21645515.2016.1215393 27715409 PMC5215426

[55] Razzaghi H, Kahn KE, Calhoun K, Garacci E, Skoff TH, Ellington SR, et al. Influenza, Tdap, and COVID-19 vaccination coverage and hesitancy among pregnant women - United States, April 2023. MMWR Morb Mortal Wkly Rep. 2023;72(39):1065–71. 37768879 10.15585/mmwr.mm7239a4 PMC10545430

[56] Park S, Lee SH, Yaroch AL, Blanck HM. Reported changes in eating habits related to less healthy foods and beverages during the COVID-19 pandemic among US adults. Nutrients. 2022;14(3). 10.3390/nu14030526 PMC8838827 35276885

[57] Ammar A, Brach M, Trabelsi K, Chtourou H, Boukhris O, Masmoudi L, et al. Effects of COVID-19 home confinement on eating behaviour and physical activity: results of the ECLB-COVID19 international online survey. Nutrients. 2020;12(6):1583. doi: 10.3390/nu12061583 32481594 PMC7352706

[58] Costa-Font J, Vilaplana-Prieto C. Trusting the health system and COVID 19 restriction compliance. Econ Hum Biol. 2023;49:101235. doi: 10.1016/j.ehb.2023.101235 36965359 PMC9946735

[59] Taylor LA, Nong P, Platt J. Fifty years of trust research in health care: a synthetic review. Milbank Q. 2023;101(1):126–78. 36689251 10.1111/1468-0009.12598 PMC10037697

[60] Daly M, Jones A, Robinson E. Public trust and willingness to vaccinate against COVID-19 in the US from October 14, 2020, to March 29, 2021. JAMA. 2021;325(23):2397–9. 34028495 10.1001/jama.2021.8246 PMC8145162

[61] Yum S. Social network analysis for coronavirus (COVID-19) in the United States. Soc Sci Q. 2020;101(4):1642–7. doi: 10.1111/ssqu.12808 32836475 PMC7283848

[62] Bayram AB, Shields T. Who trusts the WHO? Heuristics and Americans’ trust in the World Health Organization during the COVID-19 pandemic. Soc Sci Q. 2021;102(5):2312–30. doi: 10.1111/ssqu.12977 34226772 PMC8242889

[63] Center PR. Internet, broadband fact sheet. Pew Research Center; 2024.

[64] Bhandari A, Wagner T. Self-reported utilization of health care services: improving measurement and accuracy. Med Care Res Rev. 2006;63(2):217–35. doi: 10.1177/1077558705285298 16595412

[65] Doty AMB, Powell RE, Carr BG, Nelson DB, Rising KL. Identification of approaches to improve patient trust in health systems: a group concept mapping study. J Healthc Manag. 2018;63(5):e116–29. doi: 10.1097/JHM-D-17-00037 30180038

[66] Knowles M, Crowley AP, Vasan A, Kangovi S. Community health worker integration with and effectiveness in health care and public health in the United States. Annu Rev Public Health. 2023;44:363–81. 37010928 10.1146/annurev-publhealth-071521-031648

[67] Lee TH, McGlynn EA, Safran DG. A framework for increasing trust between patients and the organizations that care for them. JAMA. 2019;321(6):539–40. doi: 10.1001/jama.2018.19186 30676628

