# Supplementary material for: Trust or money? Barriers to health and healthcare behavior during the COVID-19 pandemic
Source: PLoS One. 2025 Sep 10;20(9):e0331600. doi: 10.1371/journal.pone.0331600 (PMC12422460; doi:10.1371/journal.pone.0331600)
Supplement: S1 File — Questions asked on behalf of the research team by Ipsos Online Global Omnibus in July 10–14, 2020 and May 24–29, 2023. (PDF) [file pone.0331600.s001.pdf]

S1 File. Study survey. Questions asked on behalf of the research team by Ipsos Online Global Omnibus in both July 10-14, 2020 and May 24-29, 2023.

** Signifies that there were slight changes in wording between 2020 and 2023 surveys. Both wordings are included here. The 2020 survey was also run in Spain, Italy and the United Kingdom so the original language was more international (e.g. GP rather than doctor; national rather than federal) and the 2023 survey was US-only.

Q3. How is your physical health (e.g., pain, disease) in general? How would you say it **is now**?
SINGLE CODE PER ROW, FORWARD REVERSE COLUMNS 1-5

1. Very good
2. Good
3. Fair
4. Poor
5. Very poor
6. Don't know
7. Prefer not to say

Q4. How is your mental health in general (e.g., anxiety, depression, stress, eating disorders)? How would you say it **is now**?
SINGLE CODE PER ROW, FORWARD REVERSE COLUMNS 1-5

1. Very good
2. Good
3. Fair
4. Poor
5. Very poor
6. Don't know
7. Prefer not to say

****Q8.** 2023- To what extent do you trust or not trust the way the following have dealt with the COVID-19 pandemic?

2020 - To what extent do you trust or not trust the way the following are dealing with the COVID-19 pandemic?

PROGRESSIVE GRID, FORWARD REVERSE COLUMNS 1-4, ANCHOR 5 COLUMNS

1. Trust a great deal
2. Trust a fair amount
3. Do not trust very much
4. Do not trust at all
5. Don't Know

ROWS, RANDOMISE, SINGLE CODE PER ROW

1. Your national government officials
2. Your local government officials
3. The healthcare system
4. The European Union

5. World Health Organization (the WHO)
6. Biden administration – only in 2023
7. Trump administration – only in 2023

****Q9.** 2023 - Which, if any, of the following would you say best describes your household finances?
SINGLE RESPONSE, FORWARD/REVERSE SCALE, ANCHOR CODES 6 & 7

1. Our household finances now are much better than before the COVID-19 pandemic
2. Our household finances now are a little better than before the COVID-19 pandemic
3. Our household finances now are a little worse than before the COVID-19 pandemic
4. Our household finances now are much worse than before the COVID-19 pandemic
5. There is no difference in our household finances to before the COVID-19 pandemic
6. Don't know
7. Prefer not to say

2020 - Which, if any,of the following would you say best describes your household finances during the COVID-19 lockdown?

1. Our household finances now are much better than before lockdown/stay at home order sometimes called lockdown
2. Our household finances now are a little better than before lockdown
3. Our household finances now are a little worse before lockdown
4. Our household finances now are much worse than before lockdown
5. There is no difference in our household finances to before lockdown
6. Don't know (FIX POSITION)
7. Prefer not to say

Q12. Since the outbreak of COVID – 19 are you more or less likely to do each of the following than you were before the outbreak, or is there no difference?
PROGRESSIVE GRID

COLUMNS, FORWARD REVERSE COLUMNS 1-5, ANCHOR 6-8
1. Much more likely
2. Somewhat more likely
3. No difference
4. Somewhat less likely
5. Much less likely
6. Not applicable – do not do this
7. Don't know
8. Prefer not to say

ROWS, RANDOMISE, SINGLE CODE PER ROW
1. Have a seasonal influenza vaccination
2. Have an annual health check with your doctor/ GP
3. A consultation with your doctor/healthcare professional over the telephone

4. A virtual/ remote consultation with your doctor/healthcare professional over the Internet (e.g., e-health, telehealth, etc.)
5. Use pharmacy-based health care services
6. Eat healthily
7. Exercise
8. Use digital health tools for personal health management (e.g., apps, heart rate monitor via watch)

****Q1.** 2023 - As far as you know, have you or a close family member or friend not in your household, had COVID-19?
COLLAPSABLE GRID, SINGLECODE PER COLUMN, FORWARD REVERSE ROWS 1-2,
COLUMNS
1. Yes – tested positive for COVID-19
2. No – not had COVID-19
3. Prefer not to say

ROWS RANDOMISE, SINGLECODE PER ROW
1. You
2. Close family member or friend not in your household

2020 - Q1. is As far as you know, have you or someone in your household, or a close family member or friend not in your household, had COVID-19? Please choose all that apply

1.Yes –tested positive for COVID-19 and had no or mild symptoms
2.Yes –Not tested, but think I/theyhad COVID-19 and had no or mild symptoms
3.Yes –tested positive for COVID-19 and had moderate symptoms
4.Yes –Not tested, but think I/they had COVID-19 and had moderate symptoms
5.Yes –tested positive for COVID-19 and had serious symptoms
6.Yes –Not tested, but think I/they had COVID-19 and had serious symptoms
7.Yes –died from Covid-19 (only show for 2)
8.No –not had COVID-19
9.Prefer not to say

In 2023 only **- SINTRO. The next section is about your attitude towards health.

This research is conducted in compliance with, **MRS**, **ESOMAR**, **BHBIA**, **EphMRA** codes of conduct.

Your participation is voluntary, and your responses will only be used for **market research purposes** and will not be passed to any other organisation. Your answers will be treated in the strictest confidence and results will only be reported in aggregated form, when combined with responses from others taking part. Your personal data will be held for no longer than 12 months.

In this section, we will be asking you questions about your behaviour and attitudes towards your general health as well as specific questions about Covid-19.

As always, you have the right to withdraw from the survey at any time and there will be a Prefer not to say at each question.

Are you happy to proceed with the next section on this basis?

1. Yes, I wish to continue **[CONTINUE]**
2. No, I do not wish to continue **[SKIP TO NEXT SECTION]**

****Q10.** 2023**-** **'**How likely or unlikely would you personally be to get the following vaccines in the fall/winter 2023?'

2020- 'How likely or unlikely would you personally be to get the following vaccines in the fall/autumn 2020 / winter 2020/2021 (September 2020 –March 2021), assuming they were available?'

PROGERSSIVE GRID, FORWARD REVERSE COLUMNS 1-4, ANCHOR 5 & 6 COLUMNS

1. Very likely
2. Fairly likely
3. Not very likely
4. Not at all likely
5. Don't know

ROWS, RANDOMISE, SINGLECODE PER ROW

1. **2023- COVID-19 booster (if recommended for annual seasonal boosters)
   2020 - 1. COVID-19 (if this vaccination was available)

2. Seasonal Flu (Influenza)

The next couple of questions ask about the likelihood of you and others becoming ill. Please think about each of the scenarios and provide your answer.

Q2. What would you say the risk is of you personally becoming infected with the following types of illness in the next 12 months?

Please provide your answer on a scale from 0 to 100, where 0 means there is no risk at all and you are certain **you** will not become ill and 100 means you feel extremely at risk and are certain **you** will personally become ill.
SLIDERS 0-100, LABEL 0 AS 'No risk at all, certain to not become ill', LABEL 100 as 'Extremely at risk, Certain to become ill'. ALLOW DON'T KNOW OPTION

1. COVID-19
2. Seasonal Flu (Influenza)
3. Food poisoning

Q2.1 What would you say the risk is of **an average resident of the USA,** becoming infected with the following types of illness in the next 12 months?

Please provide your answer on a scale from 0 to 100, where 0 means there is no risk at all and **the average person in the USA** will not become ill, and 100 means **the average person in the USA,** is extremely at risk and is certain to become ill..
SLIDERS 0-100, LABEL 0 AS 'No risk at all, will not become ill', LABEL 100 as 'Extremely at risk, Certain to become ill'. ALLOW DON'T KNOW OPTION

1. COVID-19
2. Seasonal Flu (Influenza)
3. Food poisoning

Q7. If you were asked to advise the government on choosing between prioritizing immediate economic gains or immediate public health gains in your country, what would be your position ~~be~~ on a 0 to 100 scale?

FORWARD REVERSE SCALE
SLIDERS 0-100, LABEL 0 AS 'Immediate economic gains' and 100 as 'Immediate public health gains'
SLIDERS 0-100, LABEL 0 AS 'Immediate public health gains' and 100 as 'Immediate economic gains'

ALLOW DON'T KNOW OPTION
