# Supplementary material for: Trust or money? Barriers to health and healthcare behavior during the COVID-19 pandemic
Source: PLoS One. 2025 Sep 10;20(9):e0331600. doi: 10.1371/journal.pone.0331600 (PMC12422460; doi:10.1371/journal.pone.0331600)
Supplement: S2 Table — (PDF) [file pone.0331600.s003.pdf]

S3 Table. Stepwise ordinary least squares regression for phone consultation with doctor.

	Phone Consultation with Doctor				
	Controls	*Adding year*	*Adding health status*	*Adding trust*	*Adding finances*
	Coef. (Std. Err.)	Coef. (Std. Err.)	Coef. (Std. Err.)	Coef. (Std. Err.)	Coef. (Std. Err.)
**Region (ref=New England)**					
Middle Atlantic	-0.046	-0.046	-0.046	-0.038	-0.049
	(0.093)	(0.079)	(0.079)	(0.095)	(0.088)
East North Central	-0.244*	-0.244**	-0.243**	-0.218*	-0.225**
	(0.095)	(0.083)	(0.080)	(0.098)	(0.083)
West North Central	-0.092	-0.092	-0.086	-0.059	-0.077
	(0.101)	(0.089)	(0.089)	(0.114)	(0.091)
South Atlantic	-0.110	-0.108	-0.107	-0.090	-0.101
	(0.087)	(0.075)	(0.076)	(0.090)	(0.081)
East South Central	-0.206	-0.203*	-0.207*	-0.181	-0.195*
	(0.105)	(0.103)	(0.089)	(0.110)	(0.096)
West South Central	-0.115	-0.116	-0.117	-0.088	-0.093
	(0.102)	(0.081)	(0.079)	(0.093)	(0.078)
Mountain	-0.133	-0.130	-0.127	-0.079	-0.095
	(0.098)	(0.096)	(0.101)	(0.110)	(0.080)
Pacific	-0.106	-0.105	-0.104	-0.101	-0.116
	(0.092)	(0.068)	(0.091)	(0.092)	(0.087)
**Age range (ref=65-75)**					
18-24	0.204*	0.188*	0.205**	0.247**	0.234**
	(0.094)	(0.091)	(0.075)	(0.087)	(0.081)
25-34	0.202*	0.192*	0.199**	0.221**	0.209**
	(0.088)	(0.082)	(0.071)	(0.075)	(0.070)
35-44	0.160*	0.151	0.146*	0.174*	0.163*
	(0.078)	(0.088)	(0.067)	(0.074)	(0.066)
45-54	0.137	0.124	0.118	0.182**	0.181**
	(0.082)	(0.079)	(0.069)	(0.063)	(0.062)
55-64	0.010	0.004	0.005	0.043	0.039
	(0.078)	(0.071)	(0.068)	(0.074)	(0.063)
**Gender (ref=Male)**					
Female	0.049	0.048	0.046	0.080*	0.080
	(0.040)	(0.037)	(0.036)	(0.038)	(0.041)

**Household income (ref=Prefer not to say)**					
\$0-\$24,999	-0.004	0.011	-0.006	-0.009	-0.036
	(0.088)	(0.102)	(0.102)	(0.092)	(0.102)
\$25,000-\$49,999	0.003	0.0132	0.001	-0.004	-0.029
	(0.092)	(0.095)	(0.104)	(0.088)	(0.101)
\$50,000-\$74,999	-0.028	-0.021	-0.025	-0.036	-0.051
	(0.097)	(0.100)	(0.104)	(0.082)	(0.086)
\$75,000-\$99,999	0.066	0.071	0.073	0.057	0.043
	(0.096)	(0.095)	(0.102)	(0.089)	(0.101)
\$100,000-\$149,999	-0.027	-0.019	-0.019	-0.048	-0.054
	(0.090)	(0.093)	(0.094)	(0.091)	(0.104)
\$150,000-\$249,999	0.148	0.151	0.165	0.110	0.108
	(0.105)	(0.110)	(0.119)	(0.104)	(0.113)
\$250,000+	0.206	0.223	0.235	0.236	0.220
	(0.179)	(0.177)	(0.187)	(0.157)	(0.166)
**Education (ref=Professional or Doctorate degree)**					
Below HS	-0.532***	-0.513***	-0.487**	-0.401*	-0.405**
	(0.151)	(0.145)	(0.165)	(0.173)	(0.155)
GED or HS diploma	-0.474***	-0.460***	-0.456***	-0.350***	-0.347***
	(0.114)	(0.096)	(0.108)	(0.094)	(0.100)
Some college	-0.357***	-0.348***	-0.349**	-0.280**	-0.281**
	(0.106)	(0.100)	(0.109)	(0.099)	(0.089)
AS degree	-0.421***	-0.416***	-0.415***	-0.327**	-0.326**
	(0.108)	(0.100)	(0.108)	(0.111)	(0.104)
BS degree	-0.408***	-0.408***	-0.403***	-0.343***	-0.340***
	(0.105)	(0.096)	(0.099)	(0.104)	(0.101)
MS degree	-0.326**	-0.322**	-0.315**	-0.295**	-0.290**
	(0.108)	(0.104)	(0.103)	(0.102)	(0.097)
**Marital status (ref=Divorced or separated)**					
Single, never married	-0.073	-0.067	-0.069	-0.053	-0.052
	(0.065)	(0.069)	(0.066)	(0.074)	(0.063)
Living with partner	-0.095	-0.087	-0.094	-0.057	-0.065
	(0.091)	(0.089)	(0.093)	(0.084)	(0.084)

Married	-0.100	-0.103	-0.094	-0.084	-0.091
	(0.058)	(0.064)	(0.073)	(0.061)	(0.054)
Widowed	-0.162	-0.164	-0.153	-0.135	-0.129
	(0.123)	(0.125)	(0.120)	(0.121)	(0.102)
**Children in household (ref=Does not have children)**					
Has children	0.082	0.091	0.096	0.073	0.062
	(0.048)	(0.053)	(0.052)	(0.045)	(0.049)
**Residence rurality (ref=Rural)**					
Urban	0.029	0.030	0.023	-0.007	-0.011
	(0.056)	(0.058)	(0.051)	(0.059)	(0.054)
**Year (ref=2020)**					
2023		-0.110**	-0.115**	-0.100**	-0.125**
		(0.039)	(0.041)	(0.035)	(0.040)
**Self-reported physical health (ref=Very good or good)**					
Fair			0.062	0.076	0.073
			(0.049)	(0.050)	(0.048)
Poor or very poor			0.170	0.166	0.166
			(0.101)	(0.098)	(0.091)
**Self-reported mental health (ref=Very good or good)**					
Fair			0.059	0.084	0.074
			(0.050)	(0.054)	(0.043)
Poor or very poor			-0.028	0.054	0.043
			(0.079)	(0.067)	(0.065)
**Trust in federal government (ref=Trust a great deal)**					
Trust a fair amount				-0.061	-0.054
				(0.083)	(0.075)
Do not trust very much				-0.058	-0.053
				(0.086)	(0.077)
Do not trust at all				0.013	0.013
				(0.092)	(0.084)

**Trust in local government (ref=Trust a great deal)**					
Trust a fair amount				-0.029	-0.028
				(0.065)	(0.067)
Do not trust very much				-0.105	-0.100
				(0.072)	(0.080)
Do not trust at all				-0.223*	-0.222*
				(0.091)	(0.093)
**Trust in the healthcare system (ref=Trust a great deal)**					
Trust a fair amount				-0.235***	-0.236***
				(0.056)	(0.052)
Do not trust very much				-0.265***	-0.265***
				(0.070)	(0.068)
Do not trust at all				-0.485***	-0.491***
				(0.099)	(0.092)
**Trust in the World Health Organization (ref=Trust a great deal)**					
Trust a fair amount				-0.116*	-0.108*
				(0.047)	(0.054)
Do not trust very much				-0.228***	-0.221***
				(0.055)	(0.059)
Do not trust at all				-0.247***	-0.249***
				(0.069)	(0.071)
**Household finances (ref=Much better)**					
A little better					-0.057
					(0.103)
A little worse					-0.072
					(0.088)
Much worse					0.010
					(0.097)
No difference					-0.168
					(0.095)
Constant	3.922***	3.986***	3.962***	4.316***	4.451***
	(0.167)	(0.170)	(0.155)	(0.173)	(0.181)

Wald x2 (p-value)	102.71 (0.000)	157.09 (0.000)	183.82 (0.000)	624.37 (0.000)	562.34 (0.000)
R2	0.028	0.031	0.037	0.089	0.093
Observations	2757	2757	2757	2757	2757

Standard errors in parentheses
* p<0.05, ** p<0.01, *** p<0.001
