# Supplementary material for: Trust or money? Barriers to health and healthcare behavior during the COVID-19 pandemic
Source: PLoS One. 2025 Sep 10;20(9):e0331600. doi: 10.1371/journal.pone.0331600 (PMC12422460; doi:10.1371/journal.pone.0331600)
Supplement: S3 Table — (PDF) [file pone.0331600.s004.pdf]

S4 Table. Stepwise ordinary least squares regression for virtual consultation with doctor.

	Virtual / Remote Consultation with Doctor				
	Controls	*Adding year*	*Adding health status*	*Adding trust*	*Adding finances*
	Coef. (Std. Err.)	Coef. (Std. Err.)	Coef. (Std. Err.)	Coef. (Std. Err.)	Coef. (Std. Err.)
**Region (ref=New England)**					
Middle Atlantic	-0.007	-0.009	-0.006	0.010	0.001
	(0.098)	(0.094)	(0.101)	(0.094)	(0.104)
East North Central	-0.099	-0.100	-0.096	-0.062	-0.068
	(0.106)	(0.093)	(0.099)	(0.084)	(0.100)
West North Central	0.039	0.036	0.044	0.088	0.076
	(0.114)	(0.105)	(0.109)	(0.112)	(0.100)
South Atlantic	-0.041	-0.044	-0.035	-0.009	-0.017
	(0.096)	(0.094)	(0.093)	(0.096)	(0.089)
East South Central	0.027	0.027	0.027	0.075	0.061
	(0.112)	(0.112)	(0.121)	(0.111)	(0.109)
West South Central	-0.077	-0.078	-0.073	-0.023	-0.028
	(0.099)	(0.091)	(0.096)	(0.103)	(0.092)
Mountain	0.010	0.011	0.016	0.068	0.057
	(0.119)	(0.104)	(0.110)	(0.100)	(0.110)
Pacific	-0.006	-0.008	-0.003	0.008	-0.004
	(0.097)	(0.090)	(0.097)	(0.090)	(0.104)
**Age range (ref=65-75)**					
18-24	0.259**	0.249**	0.262**	0.274**	0.253**
	(0.086)	(0.085)	(0.084)	(0.092)	(0.085)
25-34	0.249**	0.241***	0.250**	0.262***	0.246**
	(0.078)	(0.069)	(0.086)	(0.078)	(0.077)
35-44	0.192*	0.186**	0.183*	0.192*	0.178*
	(0.080)	(0.072)	(0.077)	(0.082)	(0.082)
45-54	0.186*	0.178*	0.174*	0.214**	0.211**
	(0.073)	(0.070)	(0.073)	(0.071)	(0.079)
55-64	0.043	0.039	0.041	0.064	0.061
	(0.068)	(0.074)	(0.066)	(0.066)	(0.073)
**Gender (ref=Male)**					
Female	0.083	0.083*	0.081	0.110**	0.111**
	(0.044)	(0.039)	(0.047)	(0.042)	(0.042)
**Household income (ref=Prefer not to say)**					

$0-$24,999	-0.026	-0.014	-0.024	-0.032	-0.060
	(0.116)	(0.113)	(0.123)	(0.123)	(0.118)
$25,000-$49,999	0.077	0.088	0.081	0.066	0.041
	(0.107)	(0.111)	(0.123)	(0.112)	(0.115)
$50,000-$74,999	-0.072	-0.063	-0.064	-0.078	-0.099
	(0.117)	(0.112)	(0.115)	(0.105)	(0.120)
$75,000-$99,999	0.006	0.012	0.013	-0.002	-0.023
	(0.111)	(0.106)	(0.122)	(0.111)	(0.107)
$100,000-$149,999	-0.015	-0.007	-0.005	-0.031	-0.045
	(0.112)	(0.111)	(0.122)	(0.111)	(0.122)
$150,000-$249,999	0.116	0.120	0.130	0.070	0.059
	(0.143)	(0.131)	(0.132)	(0.121)	(0.133)
$250,000+	0.302	0.317	0.327	0.323	0.303
	(0.191)	(0.190)	(0.212)	(0.199)	(0.210)
**Education (ref=Professional or Doctorate degree)**					
Below HS	-0.441*	-0.430*	-0.418**	-0.349	-0.356
	(0.173)	(0.191)	(0.159)	(0.181)	(0.189)
GED or HS diploma	-0.419***	-0.410***	-0.407***	-0.301*	-0.294**
	(0.107)	(0.096)	(0.094)	(0.118)	(0.097)
Some college	-0.231*	-0.225*	-0.229*	-0.174	-0.172
	(0.102)	(0.095)	(0.097)	(0.101)	(0.095)
AS degree	-0.383***	-0.379***	-0.379***	-0.303**	-0.303**
	(0.095)	(0.109)	(0.109)	(0.102)	(0.107)
BS degree	-0.225**	-0.225*	-0.225*	-0.174	-0.172*
	(0.085)	(0.090)	(0.095)	(0.092)	(0.087)
MS degree	-0.219*	-0.217*	-0.212*	-0.200	-0.197*
	(0.100)	(0.106)	(0.099)	(0.106)	(0.094)
**Marital status (ref=Divorced or separated)**					
Single, never married	0.026	0.030	0.029	0.043	0.039
	(0.070)	(0.065)	(0.069)	(0.072)	(0.073)
Living with partner	0.143	0.148	0.139	0.173	0.167
	(0.087)	(0.080)	(0.084)	(0.090)	(0.090)
Married	0.020	0.017	0.022	0.044	0.036
	(0.068)	(0.058)	(0.062)	(0.070)	(0.073)
Widowed	-0.075	-0.075	-0.070	-0.065	-0.056
	(0.127)	(0.140)	(0.137)	(0.137)	(0.129)

**Children in household (ref=Does not have children)**					
Has children	0.108*	0.115*	0.117	0.100*	0.092
	(0.047)	(0.057)	(0.061)	(0.048)	(0.050)
**Residence rurality (ref=Rural)**					
Urban	0.131	0.131**	0.127	0.101	0.099
	(0.067)	(0.050)	(0.065)	(0.063)	(0.056)
**Year (ref=2020)**					
2023		-0.077	-0.080	-0.075	-0.094*
		(0.046)	(0.046)	(0.041)	(0.044)
**Self-reported physical health (ref=Very good or good)**					
Fair			0.031	0.044	0.045
			(0.055)	(0.054)	(0.059)
Poor or very poor			0.121	0.119	0.126
			(0.107)	(0.101)	(0.095)
**Self-reported mental health (ref=Very good or good)**					
Fair			0.049	0.068	0.065
			(0.057)	(0.054)	(0.052)
Poor or very poor			-0.010	0.061	0.063
			(0.083)	(0.074)	(0.077)
**Trust in federal government (ref=Trust a great deal)**					
Trust a fair amount				0.066	0.071
				(0.075)	(0.083)
Do not trust very much				-0.022	-0.020
				(0.079)	(0.097)
Do not trust at all				0.099	0.104
				(0.079)	(0.099)
**Trust in local government (ref=Trust a great deal)**					
Trust a fair amount				-0.079	-0.079
				(0.066)	(0.065)
Do not trust very much				-0.014	-0.008
				(0.075)	(0.076)
Do not trust at all				-0.207*	-0.203*
				(0.095)	(0.097)

**Trust in the healthcare system (ref=Trust a great deal)**					
Trust a fair amount				-0.266***	-0.267***
				(0.052)	(0.055)
Do not trust very much				-0.277***	-0.274***
				(0.066)	(0.073)
Do not trust at all				-0.420***	-0.423***
				(0.094)	(0.098)
**Trust in the World Health Organization (ref=Trust a great deal)**					
Trust a fair amount				-0.075	-0.069
				(0.054)	(0.061)
Do not trust very much				-0.234***	-0.227**
				(0.056)	(0.072)
Do not trust at all				-0.282***	-0.283***
				(0.072)	(0.081)
**Household finances (ref=Much better)**					
A little better					-0.025
					(0.097)
A little worse					-0.051
					(0.085)
Much worse					-0.060
					(0.097)
No difference					-0.155*
					(0.076)
Constant	3.519***	3.564***	3.545***	3.836***	3.965***
	(0.181)	(0.153)	(0.182)	(0.169)	(0.168)
Wald x2 (p-value)	182.73 (0.000)	137.07 (0.000)	232.39 (0.000)	604.99 (0.000)	893.12 (0.000)
R2	0.035	0.036	0.040	0.085	0.088
Observations	2670	2670	2670	2670	2670

Standard errors in parentheses
* p<0.05, ** p<0.01, *** p<0.001
