# Supplementary material for: Trust or money? Barriers to health and healthcare behavior during the COVID-19 pandemic
Source: PLoS One. 2025 Sep 10;20(9):e0331600. doi: 10.1371/journal.pone.0331600 (PMC12422460; doi:10.1371/journal.pone.0331600)
Supplement: S4 Table — (PDF) [file pone.0331600.s005.pdf]

S5 Table. Stepwise ordinary least squares regression for pharmacy-based healthcare.

	Pharmacy-Based Healthcare				
	Controls	*Adding year*	*Adding health status*	*Adding trust*	*Adding finances*
	Coef. (Std. Err.)	Coef. (Std. Err.)	Coef. (Std. Err.)	Coef. (Std. Err.)	Coef. (Std. Err.)
**Region (ref=New England)**					
Middle Atlantic	0.162	0.173*	0.180*	0.167	0.146
	(0.095)	(0.087)	(0.089)	(0.093)	(0.084)
East North Central	0.034	0.041	0.050	0.078	0.060
	(0.092)	(0.092)	(0.086)	(0.087)	(0.071)
West North Central	0.173	0.180	0.190	0.223*	0.193*
	(0.101)	(0.103)	(0.101)	(0.102)	(0.097)
South Atlantic	0.126	0.133	0.141	0.147	0.128
	(0.088)	(0.085)	(0.083)	(0.088)	(0.080)
East South Central	-0.003	0.001	0.008	0.028	0.003
	(0.110)	(0.114)	(0.117)	(0.102)	(0.083)
West South Central	0.160	0.170	0.182*	0.197	0.179*
	(0.095)	(0.100)	(0.090)	(0.101)	(0.090)
Mountain	0.102	0.106	0.113	0.146	0.125
	(0.115)	(0.099)	(0.105)	(0.104)	(0.103)
Pacific	0.098	0.101	0.112	0.106	0.077
	(0.093)	(0.094)	(0.088)	(0.089)	(0.079)
**Age Range (ref=65-75)**					
18-24	0.262***	0.292***	0.299***	0.339***	0.312***
	(0.073)	(0.083)	(0.076)	(0.079)	(0.067)
25-34	0.124*	0.145*	0.156*	0.166*	0.139*
	(0.062)	(0.068)	(0.065)	(0.065)	(0.068)
35-44	0.135	0.155*	0.164*	0.188**	0.167*
	(0.072)	(0.065)	(0.065)	(0.069)	(0.066)
45-54	0.060	0.079	0.091	0.144*	0.140*
	(0.061)	(0.055)	(0.053)	(0.058)	(0.059)
55-64	0.030	0.041	0.043	0.082	0.075
	(0.059)	(0.049)	(0.052)	(0.061)	(0.054)
**Gender (ref=Male)**					
Female	-0.094**	-0.097**	-0.094*	-0.055	-0.051
	(0.036)	(0.032)	(0.038)	(0.036)	(0.039)
**Household income (ref=Prefer not to say)**					

$0-$24,999	-0.012	-0.042	-0.042	-0.050	-0.063
	(0.081)	(0.085)	(0.094)	(0.088)	(0.091)
$25,000-$49,999	-0.020	-0.043	-0.043	-0.037	-0.039
	(0.076)	(0.085)	(0.091)	(0.087)	(0.086)
$50,000-$74,999	-0.123	-0.144	-0.148	-0.137	-0.132
	(0.078)	(0.083)	(0.083)	(0.087)	(0.086)
$75,000-$99,999	0.010	-0.006	-0.014	-0.004	-0.005
	(0.084)	(0.079)	(0.089)	(0.089)	(0.085)
$100,000-$149,999	-0.073	-0.096	-0.103	-0.109	-0.100
	(0.086)	(0.082)	(0.092)	(0.097)	(0.086)
$150,000-$249,999	-0.100	-0.112	-0.122	-0.143	-0.135
	(0.100)	(0.096)	(0.093)	(0.104)	(0.100)
$250,000+	-0.025	-0.061	-0.071	-0.067	-0.068
	(0.180)	(0.171)	(0.183)	(0.160)	(0.158)
**Education (ref=Professional or Doctorate degree)**					
Below HS	-0.290	-0.321*	-0.322	-0.182	-0.189
	(0.165)	(0.148)	(0.170)	(0.145)	(0.177)
GED or HS diploma	-0.242*	-0.264*	-0.259*	-0.101	-0.102
	(0.102)	(0.110)	(0.102)	(0.110)	(0.085)
Some college	-0.181	-0.194	-0.191	-0.062	-0.064
	(0.096)	(0.110)	(0.102)	(0.100)	(0.098)
AS degree	-0.123	-0.130	-0.132	0.024	0.021
	(0.103)	(0.112)	(0.109)	(0.104)	(0.087)
BS degree	-0.130	-0.127	-0.128	-0.004	-0.004
	(0.098)	(0.100)	(0.097)	(0.096)	(0.088)
MS degree	-0.053	-0.058	-0.059	0.010	0.012
	(0.102)	(0.110)	(0.106)	(0.104)	(0.098)
**Marital status (ref=Divorced or separated)**					
Single, never married	0.058	0.050	0.057	0.043	0.040
	(0.064)	(0.064)	(0.065)	(0.066)	(0.067)
Living with partner	0.104	0.089	0.096	0.114	0.107
	(0.073)	(0.084)	(0.073)	(0.076)	(0.086)
Married	0.125*	0.131*	0.128*	0.112	0.106
	(0.058)	(0.059)	(0.061)	(0.065)	(0.069)
Widowed	0.088	0.092	0.091	0.068	0.082
	(0.107)	(0.109)	(0.117)	(0.103)	(0.120)

**Children in household (ref=Does not have children)**					
Has children	0.115*	0.101	0.099*	0.043	0.026
	(0.053)	(0.052)	(0.049)	(0.050)	(0.049)
**Residence rurality (ref=Rural)**					
Urban	0.032	0.029	0.028	-0.010	-0.014
	(0.053)	(0.055)	(0.048)	(0.043)	(0.046)
**Year (ref=2020)**					
2023		0.193***	0.195***	0.169***	0.138***
		(0.034)	(0.036)	(0.038)	(0.040)
**Self-reported physical health (ref=Very good or good)**					
Fair			-0.005	0.013	0.014
			(0.049)	(0.048)	(0.051)
Poor or very poor			-0.039	-0.019	-0.016
			(0.086)	(0.083)	(0.081)
**Self-reported mental health (ref=Very good or good)**					
Fair			-0.055	-0.015	-0.018
			(0.044)	(0.046)	(0.044)
Poor or very poor			-0.044	0.046	0.045
			(0.062)	(0.069)	(0.060)
**Trust in federal government (ref=Trust a great deal)**					
Trust a fair amount				-0.189*	-0.177*
				(0.081)	(0.073)
Do not trust very much				-0.318***	-0.304***
				(0.090)	(0.076)
Do not trust at all				-0.336***	-0.321***
				(0.090)	(0.074)
**Trust in local government (ref=Trust a great deal)**					
Trust a fair amount				-0.033	-0.031
				(0.057)	(0.060)
Do not trust very much				-0.010	-0.001
				(0.064)	(0.071)
Do not trust at all				-0.110	-0.106
				(0.076)	(0.083)

**Trust in the healthcare system (ref=Trust a great deal)**					
Trust a fair amount				-0.181***	-0.184***
				(0.047)	(0.052)
Do not trust very much				-0.275***	-0.272***
				(0.059)	(0.062)
Do not trust at all				-0.451***	-0.453***
				(0.079)	(0.083)
**Trust in the World Health Organization (ref=Trust a great deal)**					
Trust a fair amount				-0.066	-0.057
				(0.054)	(0.055)
Do not trust very much				-0.197***	-0.186**
				(0.056)	(0.061)
Do not trust at all				-0.212**	-0.208**
				(0.065)	(0.072)
**Household finances (ref=Much better)**					
A little better					-0.082
					(0.092)
A little worse					-0.185*
					(0.085)
Much worse					-0.127
					(0.085)
No difference					-0.277**
					(0.089)
Constant	3.231***	3.113***	3.118***	3.576***	3.795***
	(0.157)	(0.160)	(0.154)	(0.197)	(0.172)
Wald x2 (p-value)	135.02 (0.000)	176.87 (0.000)	190.67 (0.000)	943.26 (0.000)	1463.06 (0.000)
R2	0.031	0.040	0.042	0.118	0.126
Observations	2681	2681	2681	2681	2681

Standard errors in parentheses
* p<0.05, ** p<0.01, *** p<0.001
