# Supplementary material for: Trust or money? Barriers to health and healthcare behavior during the COVID-19 pandemic
Source: PLoS One. 2025 Sep 10;20(9):e0331600. doi: 10.1371/journal.pone.0331600 (PMC12422460; doi:10.1371/journal.pone.0331600)
Supplement: S6 Table — (PDF) [file pone.0331600.s007.pdf]

S7 Table. Stepwise ordinary least squares regression for eating healthily.

	Eat Healthily				
	Controls	*Adding year*	*Adding health status*	*Adding trust*	*Adding finances*
	Coef. (Std. Err.)	Coef. (Std. Err.)	Coef. (Std. Err.)	Coef. (Std. Err.)	Coef. (Std. Err.)
**Region (ref=New England)**					
Middle Atlantic	0.177*	0.178*	0.184*	0.193*	0.178*
	(0.074)	(0.082)	(0.086)	(0.093)	(0.074)
East North Central	-0.029	-0.028	-0.016	-0.002	-0.014
	(0.070)	(0.077)	(0.084)	(0.080)	(0.077)
West North Central	0.067	0.064	0.072	0.093	0.077
	(0.084)	(0.091)	(0.098)	(0.095)	(0.083)
South Atlantic	0.189**	0.188*	0.187*	0.201**	0.188**
	(0.063)	(0.076)	(0.080)	(0.076)	(0.073)
East South Central	0.153	0.148	0.144	0.160	0.146
	(0.086)	(0.089)	(0.099)	(0.108)	(0.088)
West South Central	0.142	0.142	0.157	0.171	0.159*
	(0.081)	(0.081)	(0.092)	(0.089)	(0.075)
Mountain	0.222**	0.218*	0.226*	0.239**	0.229**
	(0.081)	(0.090)	(0.096)	(0.084)	(0.087)
Pacific	0.162*	0.161*	0.177*	0.172	0.147*
	(0.069)	(0.082)	(0.074)	(0.089)	(0.070)
**Age range (ref=65-75)**					
18-24	0.221**	0.252**	0.292***	0.309***	0.280***
	(0.080)	(0.078)	(0.085)	(0.077)	(0.074)
25-34	0.221***	0.243***	0.275***	0.280***	0.255***
	(0.054)	(0.065)	(0.056)	(0.060)	(0.060)
35-44	0.210**	0.229***	0.260***	0.263***	0.247***
	(0.065)	(0.068)	(0.064)	(0.058)	(0.063)
45-54	0.059	0.079	0.108*	0.129**	0.128*
	(0.060)	(0.057)	(0.055)	(0.050)	(0.056)
55-64	0.096	0.109*	0.113*	0.122*	0.116*
	(0.061)	(0.052)	(0.057)	(0.050)	(0.049)
**Gender**					
Female	-0.084**	-0.085*	-0.077*	-0.053	-0.052
	(0.031)	(0.033)	(0.033)	(0.032)	(0.032)
**Household income (ref=Prefer not to say)**					

$0-$24,999	0.117	0.092	0.097	0.069	0.045
	(0.097)	(0.084)	(0.084)	(0.064)	(0.091)
$25,000-$49,999	0.055	0.037	0.039	0.023	0.009
	(0.081)	(0.082)	(0.085)	(0.077)	(0.085)
$50,000-$74,999	0.025	0.008	-0.009	-0.027	-0.040
	(0.083)	(0.081)	(0.081)	(0.069)	(0.082)
$75,000-$99,999	0.066	0.055	0.027	0.017	0.005
	(0.091)	(0.092)	(0.086)	(0.083)	(0.087)
$100,000-$149,999	-0.041	-0.055	-0.090	-0.113	-0.117
	(0.092)	(0.087)	(0.083)	(0.085)	(0.085)
$150,000-$249,999	0.054	0.048	0.012	-0.034	-0.036
	(0.113)	(0.094)	(0.099)	(0.089)	(0.099)
$250,000+	-0.036	-0.069	-0.099	-0.093	-0.109
	(0.175)	(0.166)	(0.172)	(0.163)	(0.146)
**Education (ref=Professional or Doctorate degree)**					
Below HS	-0.267	-0.302	-0.265	-0.211	-0.207
	(0.169)	(0.166)	(0.165)	(0.156)	(0.161)
GED or HS diploma	-0.136	-0.161	-0.145	-0.082	-0.068
	(0.092)	(0.087)	(0.093)	(0.087)	(0.082)
Some college	-0.123	-0.139	-0.118	-0.073	-0.065
	(0.087)	(0.085)	(0.085)	(0.081)	(0.080)
AS degree	-0.024	-0.035	-0.025	0.036	0.044
	(0.098)	(0.090)	(0.084)	(0.086)	(0.089)
BS degree	-0.043	-0.043	-0.040	0.000	0.010
	(0.085)	(0.082)	(0.087)	(0.082)	(0.082)
MS degree	-0.035	-0.041	-0.039	-0.025	-0.013
	(0.100)	(0.088)	(0.091)	(0.077)	(0.086)
**Marital status (ref=Divorced or separated)**					
Single, never married	0.034	0.028	0.044	0.045	0.045
	(0.065)	(0.064)	(0.057)	(0.068)	(0.064)
Living with partner	-0.057	-0.070	-0.055	-0.039	-0.046
	(0.082)	(0.082)	(0.080)	(0.071)	(0.075)
Married	0.053	0.060	0.047	0.040	0.035
	(0.058)	(0.050)	(0.054)	(0.060)	(0.051)
Widowed	0.108	0.116	0.104	0.090	0.097
	(0.089)	(0.087)	(0.086)	(0.088)	(0.100)

**Children in household (ref=Does not have children)**					
Has children	0.141**	0.126**	0.125**	0.102*	0.087
	(0.045)	(0.043)	(0.045)	(0.045)	(0.046)
**Residence rurality (ref=Rural)**					
Urban	0.027	0.023	0.023	0.002	0.000
	(0.041)	(0.049)	(0.047)	(0.046)	(0.045)
**Year (ref=2020)**					
2023		0.193***	0.196***	0.179***	0.147***
		(0.029)	(0.037)	(0.036)	(0.032)
**Self-reported physical health (ref=Very good or good)**					
Fair			-0.099*	-0.086*	-0.086
			(0.043)	(0.044)	(0.048)
Poor or very poor			-0.100	-0.090	-0.086
			(0.087)	(0.078)	(0.090)
**Self-reported mental health (ref=Very good or good)**					
Fair			-0.093**	-0.070	-0.076
			(0.036)	(0.041)	(0.043)
Poor or very poor			-0.250***	-0.213***	-0.217***
			(0.064)	(0.059)	(0.065)
**Trust in federal government (ref=Trust a great deal)**					
Trust a fair amount				-0.083	-0.063
				(0.072)	(0.067)
Do not trust very much				-0.176*	-0.154*
				(0.080)	(0.070)
Do not trust at all				-0.118	-0.096
				(0.086)	(0.082)
**Trust in local government (ref=Trust a great deal)**					
Trust a fair amount				-0.037	-0.036
				(0.056)	(0.054)
Do not trust very much				-0.037	-0.032
				(0.071)	(0.064)
Do not trust at all				0.009	0.004
				(0.083)	(0.067)

**Trust in the healthcare system (ref=Trust a great deal)**					
Trust a fair amount				-0.091	-0.093
				(0.050)	(0.051)
Do not trust very much				-0.253***	-0.251***
				(0.064)	(0.061)
Do not trust at all				-0.186*	-0.189*
				(0.077)	(0.074)
**Trust in the World Health Organization (ref=Trust a great deal)**					
Trust a fair amount				-0.124*	-0.116*
				(0.053)	(0.051)
Do not trust very much				-0.172**	-0.163*
				(0.058)	(0.065)
Do not trust at all				-0.172**	-0.174**
				(0.064)	(0.067)
**Household finances (ref=Much better)**					
A little better					-0.181*
					(0.088)
A little worse					-0.164*
					(0.082)
Much worse					-0.164*
					(0.082)
No difference					-0.318***
					(0.078)
Constant	3.356***	3.246***	3.290***	3.643***	3.886***
	(0.142)	(0.140)	(0.169)	(0.150)	(0.180)
Wald x2 (p-value)	171.76 (0.000)	195.30 (0.000)	288.99 (0.000)	612.60 (0.000)	778.57 (0.000)
R2	0.034	0.044	0.058	0.085	0.094
Observations	3125	3125	3125	3125	3125

Standard errors in parentheses
* $p<0.05$, ** $p<0.01$, *** $p<0.001$
