# Supplementary material for: Trust or money? Barriers to health and healthcare behavior during the COVID-19 pandemic
Source: PLoS One. 2025 Sep 10;20(9):e0331600. doi: 10.1371/journal.pone.0331600 (PMC12422460; doi:10.1371/journal.pone.0331600)
Supplement: S7 Table — (PDF) [file pone.0331600.s008.pdf]

**S8 Table. Stepwise ordinary least squares regression for exercising.**

| | Exercise | | | | |
|---|---|---|---|---|---|
| | *Controls* | *Adding year* | *Adding health status* | *Adding trust* | *Adding finances* |
| | Coef. (Std. Err.) | Coef. (Std. Err.) | Coef. (Std. Err.) | Coef. (Std. Err.) | Coef. (Std. Err.) |
| **Region (ref=New England)** | | | | | |
| *Middle Atlantic* | 0.233* | 0.234** | 0.237** | 0.232** | 0.215* |
| | (0.095) | (0.073) | (0.080) | (0.088) | (0.095) |
| *East North Central* | 0.125 | 0.127 | 0.135 | 0.150 | 0.135 |
| | (0.093) | (0.081) | (0.077) | (0.085) | (0.086) |
| *West North Central* | 0.130 | 0.127 | 0.135 | 0.152 | 0.133 |
| | (0.107) | (0.099) | (0.098) | (0.102) | (0.105) |
| *South Atlantic* | 0.310*** | 0.310*** | 0.308*** | 0.319*** | 0.303*** |
| | (0.094) | (0.077) | (0.078) | (0.088) | (0.086) |
| *East South Central* | 0.233 | 0.231* | 0.223* | 0.234* | 0.216* |
| | (0.123) | (0.112) | (0.090) | (0.108) | (0.096) |
| *West South Central* | 0.240* | 0.242** | 0.257** | 0.269** | 0.254** |
| | (0.097) | (0.082) | (0.083) | (0.095) | (0.094) |
| *Mountain* | 0.216* | 0.211* | 0.215* | 0.220* | 0.207* |
| | (0.107) | (0.095) | (0.089) | (0.101) | (0.101) |
| *Pacific* | 0.269** | 0.271** | 0.289*** | 0.283*** | 0.259** |
| | (0.096) | (0.095) | (0.076) | (0.079) | (0.094) |
| **Age range (ref=65-75)** | | | | | |
| *18-24* | 0.425*** | 0.452*** | 0.486*** | 0.476*** | 0.451*** |
| | (0.090) | (0.074) | (0.078) | (0.089) | (0.079) |
| *25-34* | 0.233** | 0.252*** | 0.275*** | 0.256*** | 0.230*** |
| | (0.073) | (0.067) | (0.069) | (0.071) | (0.067) |
| *35-44* | 0.192** | 0.209** | 0.239*** | 0.222** | 0.206** |
| | (0.072) | (0.068) | (0.067) | (0.075) | (0.071) |
| *45-54* | 0.050 | 0.068 | 0.102 | 0.113 | 0.112 |
| | (0.059) | (0.058) | (0.054) | (0.062) | (0.059) |
| *55-64* | 0.046 | 0.056 | 0.060 | 0.064 | 0.060 |
| | (0.053) | (0.052) | (0.050) | (0.057) | (0.051) |
| **Gender** | | | | | |
| *Female* | -0.094** | -0.094** | -0.087** | -0.056 | -0.052 |
| | (0.031) | (0.035) | (0.032) | (0.035) | (0.040) |

| | | | | | |
|---|---|---|---|---|---|
| **Household income (ref=Prefer not to say)** | | | | | |
| *\$0-\$24,999* | 0.121 | 0.100 | 0.111 | 0.091 | 0.087 |
| | (0.096) | (0.091) | (0.105) | (0.091) | (0.099) |
| *\$25,000-\$49,999* | 0.113 | 0.097 | 0.101 | 0.083 | 0.092 |
| | (0.091) | (0.088) | (0.097) | (0.084) | (0.092) |
| *\$50,000-\$74,999* | 0.036 | 0.023 | 0.005 | -0.015 | -0.004 |
| | (0.083) | (0.081) | (0.100) | (0.089) | (0.092) |
| *\$75,000-\$99,999* | 0.106 | 0.096 | 0.059 | 0.048 | 0.052 |
| | (0.090) | (0.079) | (0.095) | (0.085) | (0.095) |
| *\$100,000-\$149,999* | 0.021 | 0.009 | -0.036 | -0.059 | -0.047 |
| | (0.088) | (0.087) | (0.105) | (0.089) | (0.100) |
| *\$150,000-\$249,999* | 0.029 | 0.024 | -0.031 | -0.054 | -0.043 |
| | (0.111) | (0.108) | (0.111) | (0.101) | (0.092) |
| *\$250,000+* | -0.110 | -0.140 | -0.187 | -0.166 | -0.169 |
| | (0.160) | (0.163) | (0.150) | (0.173) | (0.135) |
| **Education (ref=Professional or Doctorate degree)** | | | | | |
| *Below HS* | -0.102 | -0.128 | -0.106 | -0.023 | -0.024 |
| | (0.156) | (0.155) | (0.161) | (0.176) | (0.178) |
| *GED or HS diploma* | -0.008 | -0.029 | -0.023 | 0.040 | 0.047 |
| | (0.084) | (0.093) | (0.093) | (0.098) | (0.105) |
| *Some college* | -0.107 | -0.120 | -0.100 | -0.046 | -0.042 |
| | (0.084) | (0.084) | (0.091) | (0.096) | (0.104) |
| *AS degree* | 0.028 | 0.019 | 0.027 | 0.087 | 0.091 |
| | (0.086) | (0.094) | (0.093) | (0.110) | (0.108) |
| *BS degree* | -0.026 | -0.023 | -0.027 | 0.011 | 0.020 |
| | (0.072) | (0.084) | (0.091) | (0.093) | (0.106) |
| *MS degree* | 0.024 | 0.021 | 0.017 | 0.034 | 0.041 |
| | (0.083) | (0.084) | (0.090) | (0.096) | (0.111) |
| **Marital status (ref=Divorced or separated)** | | | | | |
| *Single, never married* | -0.027 | -0.032 | -0.010 | -0.006 | -0.011 |
| | (0.069) | (0.072) | (0.066) | (0.062) | (0.056) |
| *Living with partner* | 0.040 | 0.029 | 0.050 | 0.082 | 0.077 |
| | (0.083) | (0.080) | (0.085) | (0.081) | (0.074) |
| *Married* | 0.097 | 0.104 | 0.091 | 0.080 | 0.078 |
| | (0.063) | (0.056) | (0.063) | (0.063) | (0.057) |
| *Widowed* | 0.025 | 0.027 | 0.016 | 0.001 | 0.012 |

| | | | | | |
|---|---|---|---|---|---|
| | (0.098) | (0.103) | (0.096) | (0.106) | (0.097) |
| **Children in household (ref=Does not have children)** | | | | | |
| *Has children* | 0.135** | 0.122* | 0.121** | 0.095* | 0.079 |
| | (0.052) | (0.051) | (0.045) | (0.047) | (0.049) |
| **Residence rurality (ref=Rural)** | | | | | |
| *Urban* | 0.123* | 0.120* | 0.121* | 0.095 | 0.093 |
| | (0.055) | (0.055) | (0.055) | (0.050) | (0.050) |
| **Year (ref=2020)** | | | | | |
| *2023* | | 0.176*** | 0.184*** | 0.161*** | 0.135*** |
| | | (0.036) | (0.037) | (0.039) | (0.036) |
| **Self-reported physical health (ref=Very good or good)** | | | | | |
| *Fair* | | | -0.189*** | -0.168*** | -0.167*** |
| | | | (0.042) | (0.047) | (0.044) |
| *Poor or very poor* | | | -0.181* | -0.174* | -0.174* |
| | | | (0.091) | (0.073) | (0.088) |
| **Self-reported mental health (ref=Very good or good)** | | | | | |
| *Fair* | | | -0.118** | -0.095* | -0.092* |
| | | | (0.045) | (0.042) | (0.046) |
| *Poor or very poor* | | | -0.210*** | -0.178** | -0.174** |
| | | | (0.061) | (0.063) | (0.060) |
| **Trust in federal government (ref=Trust a great deal)** | | | | | |
| *Trust a fair amount* | | | | -0.241** | -0.222** |
| | | | | (0.079) | (0.075) |
| *Do not trust very much* | | | | -0.332*** | -0.306*** |
| | | | | (0.090) | (0.084) |
| *Do not trust at all* | | | | -0.312*** | -0.282** |
| | | | | (0.090) | (0.089) |
| **Trust in local government (ref=Trust a great deal)** | | | | | |
| *Trust a fair amount* | | | | -0.017 | -0.013 |
| | | | | (0.064) | (0.055) |
| *Do not trust very much* | | | | -0.054 | -0.048 |
| | | | | (0.075) | (0.066) |
| *Do not trust at all* | | | | -0.062 | -0.066 |
| | | | | (0.089) | (0.080) |

| | | | | | |
|---|---|---|---|---|---|
| **Trust in the healthcare system (ref=Trust a great deal)** | | | | | |
| *Trust a fair amount* | | | | -0.022 | -0.026 |
| | | | | (0.051) | (0.050) |
| *Do not trust very much* | | | | -0.078 | -0.077 |
| | | | | (0.065) | (0.064) |
| *Do not trust at all* | | | | 0.099 | 0.097 |
| | | | | (0.092) | (0.089) |
| **Trust in the World Health Organization (ref=Trust a great deal)** | | | | | |
| *Trust a fair amount* | | | | -0.094 | -0.086 |
| | | | | (0.058) | (0.052) |
| *Do not trust very much* | | | | -0.153* | -0.143* |
| | | | | (0.072) | (0.063) |
| *Do not trust at all* | | | | -0.143 | -0.138* |
| | | | | (0.076) | (0.066) |
| **Household finances (ref=Much better)** | | | | | |
| *A little better* | | | | | -0.163 |
| | | | | | (0.086) |
| *A little worse* | | | | | -0.233** |
| | | | | | (0.076) |
| *Much worse* | | | | | -0.206** |
| | | | | | (0.070) |
| *No difference* | | | | | -0.307*** |
| | | | | | (0.072) |
| Constant | 3.035*** | 2.928*** | 3.000*** | 3.410*** | 3.640*** |
| | (0.162) | (0.165) | (0.158) | (0.167) | (0.188) |
| Wald x2 (p-value) | 216.25 (0.000) | 294.65 (0.000) | 413.98 (0.000) | 850.34 (0.000) | 868.69 (0.000) |
| R2 | 0.044 | 0.051 | 0.068 | 0.092 | 0.099 |
| Observations | 3025 | 3025 | 3025 | 3025 | 3025 |

Standard errors in parentheses
* p<0.05, ** p<0.01, *** p<0.001
