# Supplementary material for: Trust or money? Barriers to health and healthcare behavior during the COVID-19 pandemic
Source: PLoS One. 2025 Sep 10;20(9):e0331600. doi: 10.1371/journal.pone.0331600 (PMC12422460; doi:10.1371/journal.pone.0331600)
Supplement: S8 Table — (PDF) [file pone.0331600.s009.pdf]

S9 Table. Weighted descriptive statistics for dependent variables, 2020 and 2023.

VARIABLES	Mean (SD) 2020	Mean (SD) 2023	Difference (95% CI); P-value
Healthcare utilization likelihood relative to before the COVID-19 pandemic			
**Annual health check**			
Much less likely or somewhat less likely	0.094 (0.292)	0.063** (0.243)	-0.31 (-0.52 to -0.01); 0.003
No difference	0.580 (0.494)	0.478*** (0.500)	-0.10 (-0.15 to -0.06); <.001
Somewhat more likely or much more likely	0.249 (0.433)	0.413*** (0.492)	0.16 (0.12 to 0.21); <.001
Not applicable	0.050 (0.217)	0.026** (0.158)	-0.02 (-0.04 to –0.01); 0.001
Don't know	0.026 (0.159)	0.015 (0.121)	-0.01 (-0.02 to –0.00); 0.050
Prefer not to say	0.001 (0.031)	0.006** (0.076)	0.00 (0.00 to 0.01); 0.009
**Phone consultation with doctor**			
Much less likely or somewhat less likely	0.053 (0.225)	0.078** (0.268)	0.02 (0.01 to 0.04); 0.007
No difference	0.337 (0.473)	0.384* (0.486)	0.05 (0.00 to 0.09); 0.032
Somewhat more likely or much more likely	0.441 (0.497)	0.411 (0.492)	-0.03 (-0.07 to 0.14); 0.182
Not applicable	0.144 (0.352)	0.102*** (0.303)	-0.04 (-0.07 to –0.02); 0.001
Don't know	0.023 (0.152)	0.018 (0.133)	-0.01 (-0.02 to 0.01); 0.314
Prefer not to say	0.002 (0.039)	0.007* (0.081)	0.01 (0.00 to 0.01); 0.017
**Virtual / remote consultation with doctor**			
Much less likely or somewhat less likely	0.062 (0.241)	0.086* (0.280)	0.02 (0.01 to 0.04); 0.013
No difference	0.259 (0.438)	0.319** (0.466)	0.06 (0.02 to 0.10); 0.002
Somewhat more likely or much more likely	0.465 (0.499)	0.447 (0.497)	-0.02 (-0.06 to 0.03); 0.420
Not applicable	0.178 (0.382)	0.120** (0.326)	-0.06 (-0.09 to –0.02); 0.001
Don't know	0.035 (0.184)	0.021* (0.143)	-0.01 (-0.03 to –0.00); 0.027
Prefer not to say	0.002 (0.043)	0.007* (0.083)	0.01 (0.00 to 0.01); 0.026
**Pharmacy-based healthcare**			

Much less likely or somewhat less likely	0.070<br>(0.256)	0.073<br>(0.260)	0.02 (-0.02 to 0.03);<br>0.837
No difference	0.491<br>(0.500)	0.452<br>(0.498)	-0.04 (-0.08 to 0.00);<br>0.075
Somewhat more likely or much more likely	0.205<br>(0.404)	0.347***<br>(0.476)	0.14 (0.10 to 0.18);<br><.001
Not applicable	0.190<br>(0.392)	0.094***<br>(0.292)	-0.10 (-0.13 to –0.07);<br><.001
Don't know	0.043<br>(0.203)	0.028*<br>(0.166)	-0.01 (-0.03 to–0.00);<br>0.046
Prefer not to say	0.001<br>(0.031)	0.006*<br>(0.076)	0.00 (0.00 to 0.01);<br>0.010
Preventive health behavior likelihood relative to before the COVID-19 pandemic			
**Seasonal influenza vaccination**			
Much less likely or somewhat less likely	0.047<br>(0.212)	0.096***<br>(0.295)	0.05 (0.03 to 0.07);<br><.001
No difference	0.504<br>(0.500)	0.422***<br>(0.494)	-0.08 (-0.13 to –0.04);<br><.001
Somewhat more likely or much more likely	0.290<br>(0.454)	0.363**<br>(0.481)	0.07 (0.03 to 0.11);<br>0.001
Not applicable	0.135<br>(0.342)	0.085***<br>(0.279)	-0.05 (-0.08 to –0.02);<br><.001
Don't know	0.023<br>(0.151)	0.026<br>(0.160)	0.00 (-0.01 to 0.01);<br>0.610
Prefer not to say	0.001<br>(0.031)	0.008**<br>(0.089)	0.01 (0.00 to 0.01);<br>0.001
**Eat healthily**			
Much less likely or somewhat less likely	0.056<br>(0.230)	0.045<br>(0.208)	-0.01 (-0.03 to 0.01);<br>0.213
No difference	0.513<br>(0.500)	0.413***<br>(0.492)	-0.10 (-0.14 to –0.06);<br><.001
Somewhat more likely or much more likely	0.398<br>(0.490)	0.508***<br>(0.500)	0.11 (0.07 to 0.15);<br><.001
Not applicable	0.021<br>(0.145)	0.019<br>(0.137)	0.00 (-0.01 to 0.01);<br>0.676
Don't know	0.010<br>(0.102)	0.010<br>(0.098)	0.00 (-0.01 to 0.01);<br>0.828
Prefer not to say	0.001<br>(0.031)	0.005*<br>(0.073)	0.00 (0.00 to 0.01);<br>0.029
**Exercise**			
Much less likely or somewhat less likely	0.102<br>(0.303)	0.060**<br>(0.237)	-0.04 (-0.07 to –0.01);<br>0.005
No difference	0.436<br>(0.496)	0.422<br>(0.494)	-0.01 (-0.06 to 0.03);<br>0.523

Somewhat more likely or much more likely	0.389<br>(0.488)	0.462**<br>(0.499)	0.07 (0.03 to 0.16);<br>0.001
Not applicable	0.056<br>(0.230)	0.036*<br>(0.186)	-0.02 (-0.04 to 0.00);<br>0.013
Don't know	0.016<br>(0.127)	0.015<br>(0.122)	0.00 (-0.01 to 0.01);<br>0.799
Prefer not to say	0.001<br>(0.031)	0.006*<br>(0.077)	0.00 (0.00 to 0.01);<br>0.010

* $p<0.05$, ** $p<0.01$, *** $p<0.001$ for difference in mean between 2023 and 2020
