# Supplementary material for: Trust or money? Barriers to health and healthcare behavior during the COVID-19 pandemic
Source: PLoS One. 2025 Sep 10;20(9):e0331600. doi: 10.1371/journal.pone.0331600 (PMC12422460; doi:10.1371/journal.pone.0331600)
Supplement: S9 Table — (PDF) [file pone.0331600.s010.pdf]

S10 Table. Weighted descriptive statistics for key study independent variables, 2020 and 2023.

VARIABLES	Mean (SD) 2020	Mean (SD) 2023	Difference (95% CI); P-value
Trust in handling of COVID-19 pandemic by stakeholders			
**Federal government**			
Trust a great deal or trust a fair amount	0.328 (0.470)	0.447*** (0.497)	0.12 (0.08 to 0.16); <.001
Do not trust very much or do not trust at all	0.638 (0.481)	0.502*** (0.500)	-0.14 (-0.18 to –0.09); <.001
Don't know	0.035 (0.183)	0.051* (0.220)	0.02 (0.00 to 0.03); 0.019
**Local government**			
Trust a great deal or trust a fair amount	0.609 (0.488)	0.563* (0.496)	-0.05 (-0.09 to –0.00); 0.035
Do not trust very much or do not trust at all	0.349 (0.477)	0.387 (0.487)	0.04 (-0.00 to 0.08); 0.077
Don't know	0.042 (0.201)	0.050 (0.219)	0.01 (-0.01 to 0.02); 0.278
**Healthcare system**			
Trust a great deal or trust a fair amount	0.633 (0.482)	0.651 (0.477)	0.02 (-0.02 to 0.06); 0.413
Do not trust very much or do not trust at all	0.324 (0.468)	0.313 (0.464)	-0.01 (-0.05 to 0.03); 0.604
Don't know	0.043 (0.202)	0.036 (0.186)	-0.01 (-0.02 to 0.01); 0.361
**World Health Organization**			
Trust a great deal or trust a fair amount	0.567 (0.496)	0.577 (0.494)	0.01 (-0.03 to 0.05); 0.646
Do not trust very much or do not trust at all	0.350 (0.477)	0.353 (0.478)	0.00 (-0.04 to 0.04); 0.897
Don't know	0.083 (0.275)	0.070 (0.255)	-0.01 (-0.04 to 0.13); 0.333
Household finances relative to before the COVID-19 pandemic			
**Household finances**			
Much better or a little better	0.164 (0.370)	0.247*** (0.431)	0.08 (0.04 to 0.12); <.001
A little worse or much worse	0.333 (0.472)	0.415*** (0.493)	0.08 (0.04 to 0.12); <.001
No difference	0.457 (0.498)	0.280*** (0.449)	-0.18 (-0.22 to –0.13); <.001
Don't know	0.031 (0.174)	0.036 (0.186)	0.00 (-0.01 to 0.02); 0.593

Prefer not to say	0.015 (0.122)	0.023 (0.149)	0.01 (-0.00 to 0.02); 0.133

* p<0.05, ** p<0.01, *** p<0.001 for difference in mean between 2023 and 2020
